# Assessment of *Agrimonia eupatoria* L. and Lipophosphonoxin (DR-6180) Combination for Wound Repair: Bridging the Gap Between Phytomedicine and Organic Chemistry

**DOI:** 10.3390/biom14121590

**Published:** 2024-12-12

**Authors:** Miriam Kaňuchová, Veronika Brindza Lachová, Kateřina Bogdanová, Jana Sabová, Petra Bonová, Tomáš Vasilenko, Ivan Kováč, Martin Novotný, Petra Mitrengová, Nitjawan Sahatsapan, Matúš Čoma, Emil Švajdlenka, Milan Kolář, Peter Bohuš, Pavel Mučaji, Robert Zajíček, Dominik Rejman, Peter Gál

**Affiliations:** 1 Department of Pharmacology, Faculty of Medicine, Pavol Jozef Šafárik University, 04001 Košice, Slovakia; miriam.kanuchova@student.upjs.sk (M.K.); jana.sabova@student.upjs.sk (J.S.); matus.coma@upjs.sk (M.Č.); 2 Department of Pharmacognosy and Botany, Faculty of Pharmacy, Comenius University, 832 32 Bratislava, Slovakia; lachova5@uniba.sk (V.B.L.); mitrengova@uniba.sk (P.M.); mucaji@fpharm.uniba.sk (P.M.); 3 Department of Microbiology, Faculty of Medicine and Dentistry, Palacký University, 779 00 Olomouc, Czech Republic; katerina.bogdanova@fnol.cz (K.B.); milan.kolar@fnol.cz (M.K.); 4 Institute of Neurobiology, Biomedical Research Center of the Slovak Academy of Sciences, 04001 Košice, Slovakia; bonova@saske.sk; 5 Department of Surgery, AGEL Hospital Košice-Šaca, Pavol Jozef Šafárik University, 04001 Košice, Slovakia; tomas.vasilenko@upjs.sk; 6 Second Department of Surgery, Louis Pasteur University Hospital, Pavol Jozef Šafárik University, 040 11 Košice, Slovakia; ivan.kovac@upjs.sk; 7 Department of Infectology and Travel Medicine, Louis Pasteur University Hospital, Pavol Jozef Šafárik University, 040 11 Košice, Slovakia; martin.novotny@upjs.sk; 8 Institute of Organic Chemistry and Biochemistry, Czech Academy of Sciences v.v.i., 160 00 Prague, Czech Republic; nitjawan.sahatsapan@uochb.cas.cz; 9 Department of Biomedical Research, East-Slovak Institute of Cardiovascular Diseases, 040 11 Košice, Slovakia; 10 Department of Chemical Theory of Drugs, Faculty of Pharmacy, Comenius University, 832 32 Bratislava, Slovakia; svajdlenka@fpharm.uniba.sk; 11 Department of Natural Drugs, Faculty of Pharmacy, Masaryk University, 601 77 Brno, Czech Republic; 12 Department of Pathology, Louis Pasteur University Hospital, Pavol Jozef Šafárik University, 040 11 Košice, Slovakia; peter.bohus@unlp.sk; 13 Prague Burn Center, Third Faculty of Medicine, University Hospital Královske Vinohrady, Charles University, 100 00 Prague, Czech Republic

**Keywords:** skin tissue, extracellular matrix, repair, regeneration, phytotherapy

## Abstract

*Agrimonia eupatoria* L. (AE) has a rich tradition of use in wound healing improvement across various cultures worldwide. In previous studies, we revealed that *Agrimonia eupatoria* L. water extract (AE) possesses a rich polyphenolic composition, displaying remarkable antioxidant properties. Our investigations also demonstrated that lipophosphonoxin (LPPO) exhibited antibacterial efficacy in vitro while preserving the proliferation and differentiation of fibroblasts and keratinocytes. Building upon our prior findings, in this study, we intended to examine whether a combination of AE and LPPO could enhance skin wound healing while retaining antibacterial attributes. The antibacterial activity of AE/LPPO against *Staphylococcus aureus* was evaluated, alongside its effects on fibroblast-to-myofibroblast transition, the formation of extracellular matrix (ECM), and endothelial cells and keratinocyte proliferation/phenotype. We also investigated AE/LPPO’s impact on TGF-*β*1 and VEGF-A signaling in keratinocytes/fibroblasts and endothelial cells, respectively. Additionally, wound healing progression in rats was examined through macroscopic observation and histological analysis. Our results indicate that AE/LPPO promotes myofibroblast-like phenotypic changes and augments ECM deposition. Clinically relevant, the AE/LPPO did not disrupt TGF-*β*1 and VEGF-A signaling and accelerated wound closure in rats. Notably, while AE and LPPO individually exhibited antibacterial activity, their combination did not lead to synergism, rather decreasing antibacterial activity, warranting further examination. These findings underscore substantial wound healing improvement facilitated by AE/LPPO, requiring further exploration in animal models closer to human physiology.

## 1. Introduction

Non-healing wounds pose a formidable challenge in healthcare, demanding innovative solutions for improved patient outcomes [1]. Recently, there has been a heightened focus on exploring the potential health benefits of phytomedicine [2,3], particularly in the context of its association with polyphenols [4,5]. Our prior research focused on *Agrimonia eupatoria* L. (AE), revealing the presence of numerous phenolic constituents in its water infusion through HPLC-MS analysis [6]. Notable compounds identified included apigenin, kaempferol, quercetin derivatives, catechin, and oligomeric proanthocyanidins, with quercetin glycosides and proanthocyanidin trimers being among the most frequently detected compounds. These flavonoids, categorized as polyphenols, have been linked to numerous beneficial biological properties in medicinal plants [6,7,8,9]. Water extract of AE has demonstrated an “insulin-like” function [10] and exhibited hepatoprotective [11] and neuroprotective properties [12], primarily attributable to their potent antioxidant properties. Motivated by those properties, we conducted an in vivo research using a rat model [6], where we unveiled the skin-protecting capabilities of AE. These findings inspired us to further study effects on various cell populations involved in wound healing in vitro, as well as on sutured incisions and open excisions in vivo [13]. AE treatment led to a myofibroblast-like phenotype, enhanced ECM deposition, and increased wound tensile strength.

In parallel, we have previously synthesized innovative compounds known as lipophosphonoxins (LPPO), which exhibit potent antibacterial activities without exhibiting toxic effects on human cells [14,15,16]. The antibacterial mechanism of action of LPPO involves cell membrane permeabilization, leading to cell death. Our research has encompassed two generations of LPPO, with the second generation displaying enhanced antibacterial properties. Furthermore, second-generation LPPOs have shown promise as additives to bone cement with antimicrobial effects [17]. Unlike typical antimicrobial drugs, LPPOs offer a distinct mechanism of action preventing microbes from developing cross-resistance [16]. This concept has been validated through a series of in vitro and in vivo experiments [18]. The combination of AE’s wound-healing properties and LPPO’s antibacterial efficacy presents a novel avenue for advancing wound care and surgical practices, which forms the basis of our investigation. Thus, the current study seeks to empirically substantiate the beneficial effects of AE in the context of skin wound repair through comprehensive experiments by bridging the gap between traditional phytomedicine and modern organic chemistry techniques (LPPO), offering potential advancements in wound treatment.

## 2. Materials and Methods

### 2.1. Agrimonia eupatoria L. (AE)

Lyophilized water infusions of *Agrimonia eupatoria* L., sourced from FYTOPHARMA, Inc. (Malacky, Slovak Republic, lot no. 793-PNY; stored at the Department of Pharmacognosy and Botany, Faculty of Pharmacy, Comenius University), were used for this study. This plant belongs to the *Rosaceae* family. The water infusions were prepared in accordance with *Pharmacopoea* *Bohemoslovaca* 4 guidelines. Specifically, 10 g of the plant’s aerial parts were infused with 100 mL of boiling water for 5 min, followed by an additional 5 min heating in a boiling water bath. After allowing the mixture to cool at room temperature for 45 min, it was filtered, frozen, and freeze-dried using the SCANVAC CoolSafe™ system (LaboGene™, Lynge, Denmark), with the lyophilization performed at −53 °C and 0.043 Pa, according to the manufacturer’s protocols.

### 2.2. Lipophosphonoxin (LPPO)

In this study, we utilized the same LPPO molecule as described in our previously published work [18]. The second-generation LPPO, DR-6180, was synthesized in multigram quantities following the method outlined in an earlier report [16].

### 2.3. Phenolics Quantification and Identification by HPLC-DAD-MS

In this study, we employed the same extract as described in our previous publication [6]. The concentration of AE was set to 10 g/L and LPPO to 10 mg/L. Compound quantification and identification, both as a mixture and separately, were carried out using an Agilent 1260 HPLC system (Agilent Technologies, Inc., Santa Clara, CA, USA) coupled with an AB Sciex Triple Quad 3500 mass spectrometer (AB Sciex Pte. Ltd., Woodlands, Singapore), equipped with an electrospray ionization (ESI) source. The separation was performed using an InfinityLab Poroshell 120 EC-C18 column (4.6 × 100 mm, 2.7 µm particle size) with an InfinityLab Poroshell 120 EC-C18 pre-column guard (4.6 × 5 mm, 2.7 µm particle size, Agilent). The column temperature was kept at 30 °C, and the diode array detector (DAD) monitored wavelengths between 190 and 600 nm.

The mobile phase consisted of solvent A (methanol with 0.1% formic acid and 1 mM ammonium formate) and solvent B (water with 0.1% formic acid and 1 mM ammonium formate). The gradient was set as follows: 0 min 10% A, 36 min 100% A, 50 min 100% A, followed by a 16 min post-run. The mobile phase flow rate was 0.3 mL/min.

The mass spectrometer operated in both positive and negative ion modes. ESI MS conditions were Q1 MS scan range of 50–1200 *m*/*z*, scan rate of 2000 Da/s, CUR gas 25, temperature of 450 °C, gas 1 at 50, gas 2 at 40, and ion spray voltage at 4500 V.

### 2.4. Transforming Growth Factor Beta 1 (TGF-β1)

In vitro experiments on human dermal fibroblasts utilized TGF-β1 (PeproTech, London, UK) at a concentration of 30 ng/mL [19] as a positive control to assess fibroblast differentiation into myofibroblasts. We investigated whether LPPO or AE influences TGF-β1 signaling, both canonical and non-canonical pathways. Simultaneously, the effects of LPPO, AE, and/or TGF-β1 were also examined in HaCaT cells to determine whether the cells would undergo epithelial–mesenchymal transition. A standard cultivation medium served as the control in all experiments.

### 2.5. Vascular Endothelial Growth Factor A (VEGF-A)

In in vitro experiments conducted on human dermal microvascular endothelial cells, VEGF-A from the Endopan MV Kit (PAN-Biotech GmbH, Aidenbach, Germany) was used as a positive control to assess the cells’ ability to undergo calcium-dependent homophilic binding at adherens junctions [20]. We investigated whether LPPO or AE influences VEGF-A signaling. The Endopan MV medium without the VEGF-A supplement was used as the control.

### 2.6. Human Keratinocyte Cell Line (HaCaT)

The HaCaT cell line [21] used in this study was obtained from Cell Lines Service (Eppelheim, Germany). The cells were cultured in Dulbecco’s Modified Eagle Medium (DMEM) supplemented with 10% fetal bovine serum (FBS) and antibiotics (streptomycin and penicillin), all sourced from Biochrom (Berlin, Germany).

### 2.7. Human Dermal Fibroblasts (HDF)

Human dermal fibroblasts were isolated from skin samples following instructions published previously [22]. Skin samples were obtained from two healthy donors undergoing aesthetic surgery, with signed informed consent in full accordance with the Helsinki Declaration and approved by the Ethical Committee of University Hospital Královské Vinohrady, Prague, Czech Republic (approval No. 100/1947/2005). The surgeries took place at the Department of Aesthetic Surgery, Third Faculty of Medicine, Charles University, Prague, Czech Republic. HDFs were cultured and expanded in DMEM supplemented with 10% FBS and ATB (penicillin/streptomycin, Biochrom, Berlin, Germany) and maintained at 37 °C with 5% CO_2_. HDFs in passages 7 or 8 were used for all experiments.

### 2.8. Human Dermal Microvascular Vein Endothelial Cells (HMVEC-d)

Dermal microvascular endothelial cells (HMVEC-d) were obtained from Lonza (Lonza Walkersville, Inc., Walkersville, MD, USA). The cells were cultured in Endopan MV, an endothelial growth medium supplemented with epidermal growth factor (EGF), fibroblast growth factor-2 (FGF-2), VEGF, ascorbic acid phosphate, R3 insulin-like growth factor 1 (R3-IGF-1), 6% FBS, gentamicin/amphotericin, and hydrocortisone (PAN-Biotech GmbH, Aidenbach, Germany). Cultures were maintained in a humidified atmosphere at 37 °C with 5% CO_2_.

### 2.9. MTS Assay Of Cultured Cells

The viability and proliferation of cells were tested using a colorimetric assay with MTS dye (3-(4,5-dimethylthiazol-2-yl)-5-(3-carboxymethoxyphenyl)-2-(4-sulfophenyl)-2H-tetrazolium) (Promega, Madison, WI, USA) [23]. Cells were seeded into 96-well plates in a culture medium containing 10% FBS at the following densities: HDFs—3000 cells/well, HaCaT—10,000 cells/well, and HMVEC-d—4000 cells/well. After 24 h, the medium was replaced with one containing LPPO, AE, or their combinations, in the presence or absence of TGF-β1 (PeproTech, London, UK) for HaCaT/HDF or VEGF-A (PAN-Biotech GmbH) for HMVEC-d cells. Seven days post-seeding, MTS (0.21 mg/mL) was added to the culture medium. After 3 h incubation, cell proliferation was measured by reading absorbance at 490 nm using the automated Cytation™ 3 Cell Imaging Multi-Mode Reader (Biotek, Winooski, VT, USA). The absorbance of the control wells was set as 100%, and the results were expressed as a percentage of the untreated control. All experiments were performed in technical triplicates and repeated three times.

### 2.10. Two-Dimensional-Migration (Wound Healing) Assay of HaCaT and HMVEC-d Cells

Cell migration of HaCaT and HMVEC-d cells was assessed using a scratch assay. To perform the assay, a confluent monolayer of cells grown in 6-well plates was scratched with a pipette tip, creating a defined “wound” area. Following the scratch, the medium was replaced with fresh medium containing varying concentrations of AE (1, 5, and 10 mg/L), LPPO (1, 5, and 10 mg/L), or a combination of AE (10 mg/L) and LPPO (5 mg/L). In the case of HaCaT cells, TGF-β1 (PeproTech, London, UK) was added, while VEGF-A (PAN-Biotech GmbH) was used for HMVEC-d cells to investigate their effects on cell migration. Images of the wounded area were captured immediately after the scratch (0 h) and at 24 h for HaCaT cells or 20 h for HMVEC-d cells. The artificial wound closure was quantified by measuring the migration distance (gap area) using NIS Elements software (Nikon, Tokyo, Japan) and expressed as a percentage of the initial wound area at 0 h. The experiments were conducted in technical duplicates and repeated three times to ensure accuracy and reproducibility.

### 2.11. Western Blot Analysis of HaCaT, HDF, and HMVEC-d Cells

Based on the results from the MTS assay, we selected non-toxic concentrations of AE (1, 5, and 10 mg/L), LPPO (1, 5, and 10 mg/L), and a combination of AE (10 mg/L) with LPPO (5 mg/L) for subsequent Western blot analysis. Protein lysates were prepared from HaCaT, HDF, and HMVEC-d cells, which were seeded in Petri dishes at densities of 10,000, 5000, and 9000 cells/cm^2^, respectively. The cells were cultivated for 7 days in the presence of LPPO or AE, along with TGF-β1 for HaCaT and HDF cells and VEGF-A for HMVEC-d cells. A comprehensive list of the primary and secondary antibodies used in this analysis is provided in Table 1. The analysis was performed following established protocols [24]. In brief, cells were washed with cold phosphate-buffered saline (PBS) and collected in Laemmli sample buffer (100 mM TRIS-HCl, pH approximately 6.8; 10% glycerol; 2% SDS), supplemented with protease and phosphatase inhibitors (Sigma-Aldrich, St. Louis, MO, USA). Immediately after collection, the cells were disrupted using a sonicator (QSonica, Newtown, CT, USA, 40% amplitude for 15 s). After sonication, the prepared cell lysates were stored at −20 °C until the next analysis. After thawing, the samples were heated at 95 °C for 5 min to denature the proteins. Subsequently, the samples were separated by SDS-PAGE on a 10% Bis-Tris gel and transferred to a PVDF membrane using the iBlot 2 system (Thermo Fisher Scientific, Inc., Waltham, MA, USA). After blocking the membranes for 1 h at room temperature in a solution of 5% non-fat dry milk/bovine serum albumin (NFDM/BSA) dissolved in TBS (tris-buffered saline) with 0.1% Tween, they were incubated overnight at 4 °C with the primary antibody. The following day, membranes were washed in TBS-Tween (3 × 5 min) and subsequently incubated with HRP-conjugated secondary antibodies for 1 h at room temperature. After another round of washing in TBS-Tween (3 × 5 min), protein bands were detected using ECL (SuperSignal West Pico PLUS chemiluminescent substrate, Thermo Fisher Scientific), and the signals were acquired using the iBright™ FL1500 Imaging System (Thermo Fisher Scientific). To ensure accurate quantification, β-actin was utilized as a loading control. The chemiluminescent signals of all detected proteins were quantified using Image Studio, Image Studio, Inc., Appleton, WI, USA, (LI-COR) Western blot densitometry software and normalized to the β-actin levels. Original figures can be found in Supplementary Materials.

### 2.12. Immunofluorescence of HaCaT and HDF Cells

HDFs and HaCaT cells were seeded at densities of 5000 and 10,000 cells/cm^2^, respectively. Both cell lines were cultivated with AE (1, 5, and 10 mg/L), LPPO (1, 5, and 10 mg/L), or a combination of AE (10 mg/L) and LPPO (5 mg/L) in the presence or absence of TGF-β1 (PeproTech, London, UK) for 7 days, using a medium containing 30 ng/mL of TGF-β1. The specific primary and secondary antibodies utilized in the analysis are listed in Table 2. The analysis was conducted following established protocols [25]. Briefly, the specimens were fixed with 2% buffered paraformaldehyde (pH 7.2) for 5 min and then washed with phosphate-buffered saline (PBS). To allow for antibody penetration, cells were permeabilized using Triton X-100 (Sigma-Aldrich, USA), and nonspecific binding sites were blocked by incubating with porcine serum albumin. Commercial antibodies were applied at the concentrations recommended by their manufacturers. After incubation with the antibodies, all specimens were mounted using Vectashield (Vector Laboratories, Eching, Germany) to preserve fluorescence. The samples were then examined using an Eclipse 90i microscope equipped with filter blocks suitable for three types of fluorescent dyes (Nikon, Tokyo, Japan), complemented by a Hamamatsu CCD camera (Hamamatsu, Shizuoka, Japan) and a computer-assisted image analysis system (NIS, Nikon, Tokyo, Japan). This setup allowed for detailed visualization and analysis of the cellular responses to the treatments.

### 2.13. Determination of MIC and MBC Values of AE and LPPO

The antimicrobial efficacy of the lyophilized water infusion of AE and LPPO DR-6180 was evaluated using the standard microdilution method to determine the minimum inhibitory concentration (MIC), following the guidelines established by the EUCAST (European Committee on Antimicrobial Susceptibility Testing). The assay was conducted in disposable 96-well microtiter plates, where the AE and LPPO were diluted in Mueller–Hinton broth (MH medium, BioRad, Hercules, CA, USA) to create concentration ranges of 2% to 0.01% for AE and 64 to 0.5 μg/mL for LPPO. Each well contained a final volume of 100 μL of the prepared suspension. A standard reference strain of *Staphylococcus aureus* CCM 4223 (ATCC 25923), sourced from the Czech Collection of Microorganisms at the Faculty of Science, Masaryk University in Brno, was utilized for the experiment. The plates were inoculated with a standardized inoculum of the test bacterium, achieving a density of 5.10^5^ CFU/mL in each well. Incubation was carried out for 24 h at a controlled temperature of 35 ± 1 °C, during which the MIC was identified as the lowest concentration of the tested compounds that visibly inhibited bacterial growth.

To further assess the bactericidal properties, the minimum bactericidal concentration (MBC) was determined by transferring 1 μL from each well, showing no visible growth onto blood agar plates (Trios, Prague, Czech Republic). These were incubated for an additional 24 h at 35 ± 1 °C. The absence of microbial colonies on the agar indicated the MBCs. Each test was performed in triplicate to ensure the accuracy and reliability of the results.

### 2.14. Determination of the Synergistic Effect of AE and LPPO

The synergistic potential of AE and LPPO was evaluated using the checkerboard microdilution method [26,27], a well-established technique for assessing the combined effects of two antimicrobial agents. This assay employs double serial dilutions, enabling the determination of each compound’s activity as well as their interactive effects when combined.

In this setup, AE was gradually diluted in Mueller–Hinton (MH) medium across the rows of a disposable microtiter plate, creating a concentration range of 2% to 0.002% (20 to 0.02 g/L), with each well containing 50 μL of the diluted suspension. Concurrently, LPPO was added vertically in 50 μL aliquots, spanning a concentration range of 32 to 0.5 mg/L. This arrangement generated a comprehensive checkerboard grid that facilitated the examination of various concentration combinations of AE and LPPO. At the same plate, the MIC determination of AE and LPPO separately, together with growth control, was performed. To ensure consistency and accuracy, the plates were inoculated with a standardized amount of the test microorganism, achieving a uniform inoculum density of 5 · 10^5^ CFU/mL in each well. Following a 24-h incubation period at 35 ± 1 °C, the determination of the minimum inhibitory concentrations (MICs) was performed for both compounds, both alone and in combination.

Subsequently, the fractional inhibitory concentrations (FICs) were calculated using the following equations:

(1) FIC_AE_ = MIC of AE in the presence of LPPO/MIC of AE alone

(2) FIC_LPPO_ = MIC of LPPO in the presence of AE/MIC of LPPO alone

The combined effect of AE and LPPO was then assessed using the sum of the FICs: ∑FICs (FIC_LPPO_ + FIC_AE_). The interpretation of the cumulative FIC values was as follows: ∑FIC ≤ 0.5 was evaluated as synergy, ∑FIC > 0.5–1 as an additive effect, ∑FIC >1 to < 2 as indifference, and ∑FIC ≥ 2 as antagonism. Each test was meticulously conducted in triplicate to ensure the robustness and reliability of the results.

### 2.15. Animal Model

The experimental conditions adhered to the European guidelines for ethical standards in animal treatment and welfare. Approval for the study was granted by the Ethics Committee of the Faculty of Pharmacy at Comenius University and the State Veterinary and Food Administration of the Slovak Republic on 28 July 2015 (Ro-2617/15-221b). Male Sprague–Dawley rats (*n* = 12), each weighing approximately 400 ± 40 g, were sourced from the Laboratory of Research Biomodels at P. J. Šafárik University for this study. The animals were housed individually in Plexiglas cages under controlled environmental conditions (55 ± 5% humidity, 22 ± 2 °C, and a 12 h light-dark cycle) and were provided unrestricted access to a standard laboratory diet and tap water.

The rats were randomly assigned to three groups, with four animals in each group. For general anesthesia, a combination of 33 mg/kg ketamine (Calypsol, Richter Gedeon, Budapest, Hungary), 11 mg/kg xylazine (Rometar a.u.v., Spofa, Prague, Czech Republic), and 5 mg/kg tramadol (Tramadol-K, Krka d.d., Novo Mesto, Slovenia) was administered via intramuscular injection. Atropine (Biotika, Slovenská Ľupča, Slovak Republic) was used for premedication at a dose of 0.05 mg/kg. Under aseptic conditions, two circular full-thickness skin excisions, each 6 mm in diameter, were made on the dorsal surface of each rat (see Figure S1). The open wounds were left undressed to facilitate natural healing. On the 14th day post-surgery, the animals were euthanized using an overdose of anesthetics, ensuring humane treatment throughout the study.

### 2.16. Wound Treatment

In the control group, wounds were left untreated, without the application of the AE/LPPO water solution. To serve as a negative control and account for the effects of wound cleansing and moist healing, the wounds in this group received daily treatment for the first three days post-surgery with LavaSurg^®^ (B. Braun, Melsungen, Germany), which was administered topically using an eye dropper.

In the experimental group, a sterile water solution containing a mixture of AE (10 g/L) and LPPO (10 mg/L) was applied topically three times a day during the initial three days following surgery. The volume of the solutions applied was carefully calibrated to ensure thorough irrigation of the wounds, promoting optimal healing conditions.

### 2.17. Basic Histology and Semi-Quantitative Scoring of Histological Sections

Wounds were systematically prepared for examination under light microscopy. The process involved several key steps: fixation in 4% buffered formaldehyde, followed by dehydration through a series of increasing alcohol concentrations. The samples were then embedded in paraffin and sectioned into 5 µm thick slices before being stained with hematoxylin-eosin (HE) for detailed visualization. To evaluate the status of re-epithelialization and the presence of polymorphonuclear leukocytes (PMNLs), fibroblasts, newly formed blood vessels, and collagen, a semi-quantitative scale was employed. This scale ranged from—(no presence) to ++++ (extensive presence), as outlined in Table 3 [28].

### 2.18. Statistical Analysis

Mean values and standard deviations (mean ± SD) were computed for all quantitative parameters to summarize the data effectively. For semi-quantitative assessments, results are depicted as medians to convey varying levels of response, utilizing a descriptive scale: − (0), + (1), ++ (2), +++ (3), and ++++ (4). To analyze the quantitative data, ANOVA was performed, followed by Turkey’s test for detailed multiple comparisons. In cases where the data did not conform to parametric assumptions, the Kruskal–Wallis test was employed to assess non-parametric semi-quantitative results. GraphPad Prism software, ver. 8.0.2.263, (GraphPad Software, San Diego, CA, USA) was used to generate fitting curves and calculate IC50 values for MTS assays. A significance threshold of *p* < 0.05 was established, ensuring that the findings were statistically relevant.

## 3. Results

### 3.1. HPLC Analysis

Using HPLC-MS, the analysis of the plant extract facilitated the identification and quantification of key phenolic compounds, mainly through their fragmentation patterns and by comparing them with reference substances. The AE extract was observed to have various phenolic compounds, mainly including derivatives of apigenin, kaempferol, and quercetin, along with catechin and oligomeric proanthocyanidins. Among the compounds identified, quercetin glycosides and proanthocyanidin trimers were the most prevalent. The lyophilized extract exhibited a total polyphenol content of around 8%, highlighting its potential as a valuable source of these phytoconstituents.

**Figure 1:**
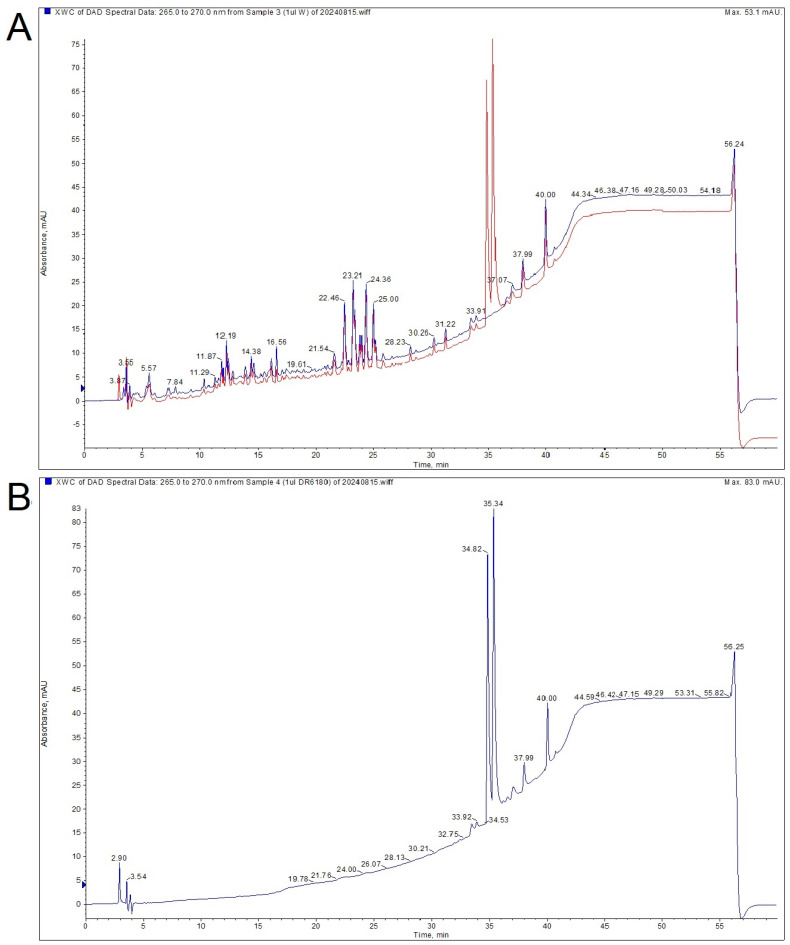
High-performance liquid chromatography (HPLC) analysis of the *Agrimonia eupatoria* L. (AE) water extract and the second-generation lipophosphonoxin (LPPO) DR-6180. (**A**) Analysis of AE (blue) and LPPO (red) mixture. (**B**) Analysis of LPPO (blue), showing two isomers represented by two distinct peaks.

During the analysis of the extract using HPLC, it was observed that the extract did not interact with LPPO DR-6180, as both the extract profile and the LPPO remained unchanged. Notably, the LPPO itself showed two distinct peaks on the HPLC, indicating the presence of two isomers (Figure 1).

### 3.2. In Vitro Experiments

Our study aimed to assess whether the AE extract, when combined with LPPO at bactericidal concentrations, influences the proliferation and differentiation of keratinocytes, fibroblasts, and endothelial cells. Additionally, we explored the capacity of the AE/LPPO combination to affect TGF-β1/VEGF-A signaling [29], which plays a crucial role in effective wound healing.

#### 3.2.1. Metabolic Assay of HaCaT, HDF, and HMVEC-d Cells Following Treatment with AE and/or LPPO

**Figure 2:**
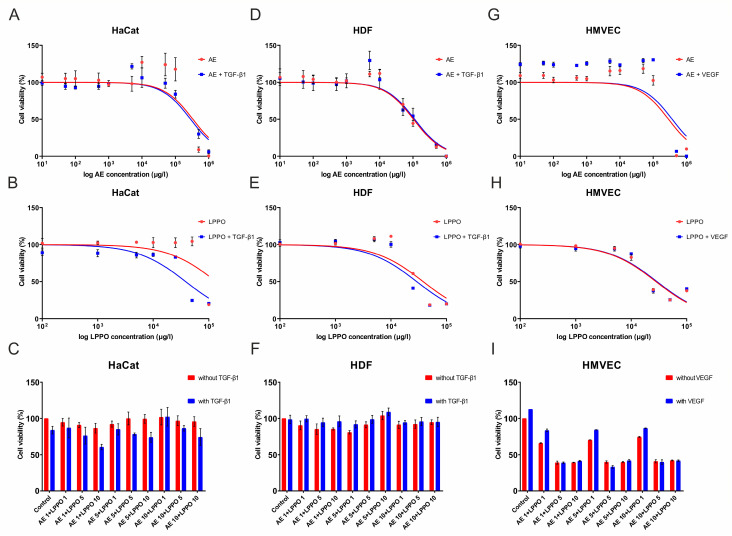
MTS assay of HaCaT keratinocytes (**A**–**C**), human dermal fibroblasts (HDF) (**D**–**F**), and human dermal microvascular vein endothelial cells (HMVEC-d) (**G**–**I**) in the presence of *Agrimonia eupatoria* L. (AE) extract, lipophosphonoxin (LPPO) DR-6180, and combination of AE and LPPO. TGF-β1 was used as the positive control in HaCaT and HDF, while VEGF-A was used in HMVEC-d. The figures were generated using GraphPad Prism software (GraphPad Software, San Diego, CA, USA), which was also utilized to logarithmically transform the concentration values and generate fitting curves to visualize the data.

The in vitro study conducted on HaCaT keratinocytes (Figure 2A–C, Table 4) demonstrated that AE at concentrations ranging from 0.01 to 100 mg/L, along with LPPO at concentrations between 0.1 and 25 mg/L (bactericidal concentrations), did not adversely affect the metabolic activity of the cells under investigation. It is noteworthy that higher tested concentrations of AE (100, 500, and 1000 mg/L) as well as LPPO (50 and 100 mg/L) showed toxicity to the studied cells.

Conversely, HDF cells (Figure 2D–F, Table 4) exhibited greater sensitivity to the compounds tested, showing a non-toxic range of 0.01 to 10 mg/L for AE and 0.1 to 10 mg/L for LPPO. HMVEC-d cells exert a similar pattern of exposure to AE than keratinocytes but exert higher sensitivity to LPPO (Figure 2G–I).

#### 3.2.2. Migration Assay of HaCaT and HMVEC-d Cells Following Treatment with AE and/or LPPO

**Figure 3:**
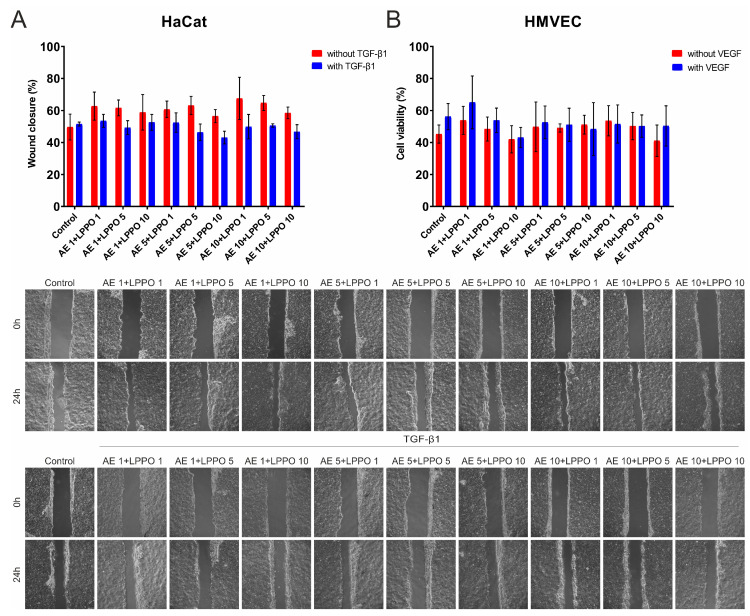
Wound healing (2D migration) assay of HaCaT keratinocytes (**A**) and human dermal microvascular vein endothelial cells—HMVEC-d (**B**) in the presence of *Agrimonia eupatoria* L. (AE) extract, lipophosphonoxin (LPPO) DR-6180, and combination of AE and LPPO. TGF-β1 was used as the positive control in HaCaT, while VEGF-A was used in HMVEC-d (Magnification 100×).

The migration assay of HaCaT cells is shown in Figure 3A and Figure S2. Keratinocyte migration was enhanced after TGF-β1 treatment (positive control) and also when treated with either AE or LPPO. However, LPPO reduced TGF-β1-induced migration at a concentration of 5 mg/L, but this effect was not observed at the lower concentrations tested. Co-treatment of cells with AE + LPPO resulted in a similar migratory pattern as that seen for the positive control. The differences between groups, however, were not statistically significant (Tables S1 and S2).

The migration assay of HMVEC-d cells is shown in Figure 3B and Figure S3. The presence of VEGF-A alone did not remarkably accelerate the migration of the HMVEC-d cells. Concentrations of LPPO higher than 1 mg/L attenuated migration of HMVEC-d cells. Treatment of the cells with AE did not influence migration. Co-treatment of cells with AE 1 mg/L and LPPO 1 mg/L accelerated the migration of cells; this was more apparent in the presence of VEGF-A. As with HaCaT cells, the differences between groups were not statistically significant (Tables S3 and S4).

#### 3.2.3. Western Blot of HaCaT, HDF, and HMVEC-d Following Treatment with AE and/or LPPO

**Figure 4:**
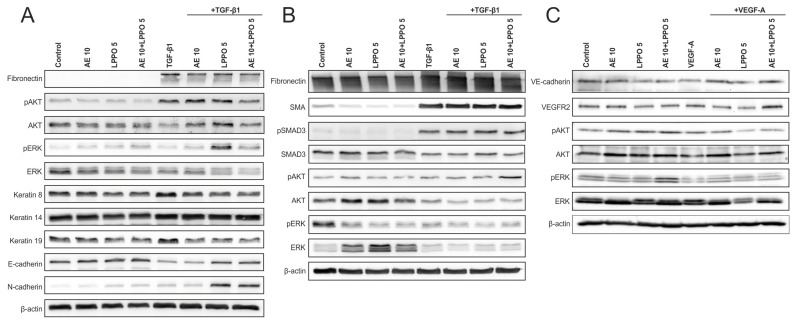
Western blot (WB) analysis of HaCaT keratinocytes (**A**), human dermal fibroblasts—HDF (**B**), and human dermal microvascular vein endothelial cells—HMVEC-d (**C**) in the presence of *Agrimonia eupatoria* L. (AE) extract, lipophosphonoxin (LPPO) DR-6180, and combination of AE and LPPO. TGF-β1 was used as the positive control in HaCaT and HDF, while VEGF-A was used in HMVEC-d.

The HaCaT phenotype (Figure 4A and Figure S4) was defined by a lack of fibronectin production, expression of total AKT/ERK forms (with minimal levels of pAKT and pERK), strong expression of keratins-8, -14, and -19, alongside the presence of E-cadherin and poor expression of N-cadherin. Treatment with TGF-β1 led to increased fibronectin and pAKT expression, a decrease in total AKT production, a slight rise in pERK levels, and a transition from E-cadherin to N-cadherin. The keratin profile remained relatively stable. AE/LPPO produced a slight stimulation of pAKT/pERK but resulted in reduced levels of total AKT and ERK. Additionally, E-cadherin expression was enhanced at higher concentrations of LPPO, while the highest concentration led to a decrease in E-cadherin levels. Notably, AE/LPPO did not significantly alter the phenotypic changes induced by TGF-β1.

The WB analysis in HDFs (Figure 4B and Figure S5) showed that TGF-β1 stimulated the canonical (SMAD) signaling, leading to enhanced SMA and fibronectin production. Additionally, TGF-β1 elevated pAKT expression, while pERK/ERK levels showed a slight decrease. Treatment of HDFs with AE/LPPO, without TGF-β1, resulted in a minor reduction in fibronectin and SMA expression. However, when TGF-β1 was present, SMA expression remained relatively stable. Notably, pAKT expression increased in the presence of both AE/LPPO and TGF-β1, whereas total AKT levels decreased. Notably, AE/LPPO did not trigger the non-canonical (pERK) signaling pathway, regardless of the presence or absence of TGF-β1. However, total ERK levels were upregulated after AE/LPPO treatment only in the absence of TGF-β1.

Experiments conducted on HMVEC-d cells (Figure 4C and Figure S6) revealed a poor expression of VEC as well as a moderate expression of the total form of VEGFR2 and a prevalence of pERK/ERK over pAKT/AKT signaling. Adding VEGF as a positive control led to an increase in VEC and a slight increase in pAKT/pERK expressions. AE/LPPO had rather a stimulatory effect on pERK than on pAKT expression. AE alone had a slight inhibitory effect on pAKT/pERK, whereas LPPO did not modulate its expression significantly. Of note, co-treatment with AE/LPPO did not significantly affect the phenotypic changes caused by VEGF.

#### 3.2.4. Immunofluorescence (IF) Analysis of HaCaT and HDF Treated with AE and LPPO

**Figure 5:**
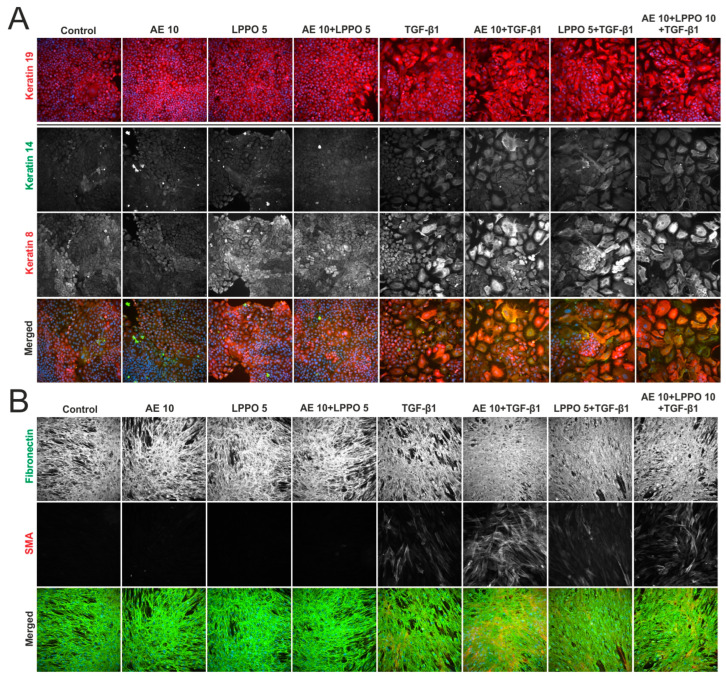
Immunofluorescence of HaCaT keratinocytes (**A**) and human dermal fibroblasts—HDF (**B**) in the presence of *Agrimonia eupatoria* L. (AE) extract, lipophosphonoxin (LPPO) DR-6180, and combination of AE and LPPO. TGF-β1 was used as the positive. Magnification 200×.

The morphology of HaCaT cells (Figure 5A and Figure S7) showed distinct differences in those treated with TGF-β1, which had larger cell sizes compared to the smaller cells in the control group. Untreated cells displayed moderate levels of keratins 14 and 19, while keratin 8 expression was marginal. Treatment with TGF-β1, LPPO, and AE/LPPO, but not AE alone, slightly increased keratin 8 levels. The phenotype changes induced by TGF-β1 were not abolished in the presence of AE, LPPO, or AE/LPPO.

IF analysis of fibronectin revealed that HDF cells (Figure 5B and Figure S8) formed an extracellular matrix (ECM), which exhibited a higher density of fibronectin after treatment with TGF-β1. As anticipated, TGF-β1 also promoted the differentiation of fibroblasts into myofibroblasts, which were characterized by well-defined SMA fibers. AE slightly induced fibronectin deposition and the rare presence of SMA-positive cells. Co-treatment of cells with AE and TGF-β1 revealed the most prominent occurrence of myofibroblasts, an effect that was only slightly reduced following the addition of LPPO.

#### 3.2.5. Antimicrobial Activity of AE and LPPO

MIC and MBC values of AE and LPPO were tested against Gram-positive *Staphylococcus aureus*. In the case of AE, the MIC was 600 mg/L, and for LPPO DR-6180, it was 4 mg/L. In both, the value of MBC was equivalent to MIC.

**Figure 6:**
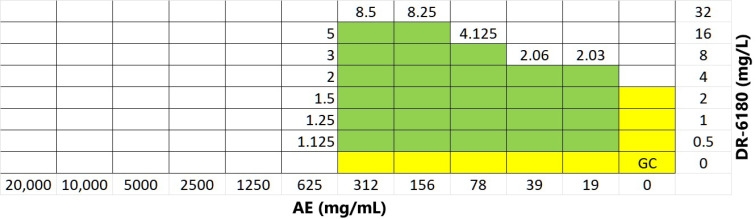
Antimicrobial effect of AE + LPPO (DR-6180) combination tested by checkerboard microdilution assay in 69-well microtiter plate. Colors indicate wells with positive bacterial growth (yellow color for growth control (GC), and AE and DR-6180 alone; green for the combinations). The numbers next to the green areas are ∑FICs (FIC_LPPO_ + FIC_AE_) values. ∑FIC >1 to < 2 is evaluated as indifference, and ∑FIC ≥ 2 is evaluated as antagonism.

To determine the possible synergy effect of combination AE + LPPO, the checkerboard microdilution assay was conducted. The results are depicted in Figure 6. Surprisingly, the effect was evaluated as antagonism. The AE elevated the MIC value of LPPO as much as 8 times (from 4 mg/L to 32 mg/L, at the concentration of AE 100 and 300 mg/L), but at the same time, LPPO did not affect the MIC of AE.

### 3.3. In Vivo Experiment

**Figure 7:**
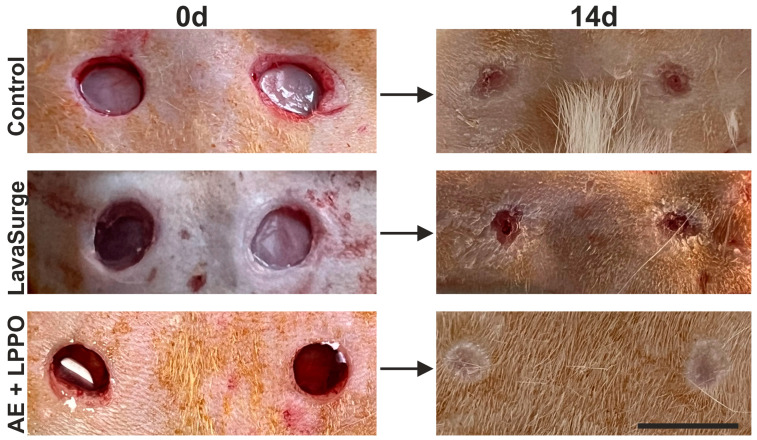
Wounds on rats from control (untreated), LavaSurg-treated (positive control, moist healing), and combination of *Agrimonia eupatoria* L. (AE) extract and lipophosphonoxin (LPPO) DR-6180 on day 14 post surgery. Scale bar = 1 cm.

We subsequently confirmed that the animal model employed is suitable for assessing the biocompatibility of the AE and LPPO mixture in treating skin wounds in rats. Specifically, during the postoperative phase, all animals remained healthy and showed no clinical signs of infection. The wound experiment was concluded, and samples were taken for histological analysis on postoperative day 14. A semi-quantitative analysis of the histological sections is summarized in Table 5. Demonstrative images of the skin wounds are displayed in Figure 7. A detailed depiction of the microscopic findings is shown below.

#### Histology of Open Wounds

**Figure 8:**
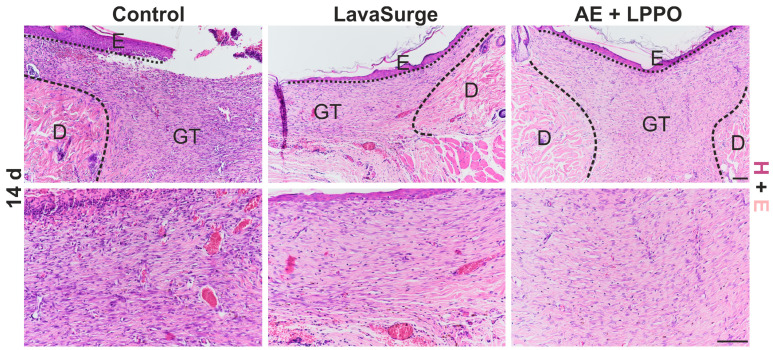
Histological assessment of wounds (hematoxylin and eosin) control (untreated), LavaSurg-treated (positive control, moist healing), and combination of *Agrimonia eupatoria* L. (AE) extract and lipophosphonoxin (LPPO) DR-6180 on day 14 post surgery. Scale bar = 100 µm.

On day 14, our observations revealed well-formed granulation tissue rich in high-caliber vessels, most visibly in the control group (Figure 8). The epidermis regeneration had not yet been fully completed in either the control group or the positive control group, which was treated with LavaSurg to eliminate the influence of moist healing. The center of the wound was still not bridged by an epithelial sheet, and the demarcation line (formed mostly of neutrophil granulocytes) separating vital tissues and scabs was still present over the granulation tissue. In contrast, wounds treated with the AE + LPPO solution demonstrated the quickest healing rate, as evidenced by the presence of a keratin layer, which signifies normal keratinocyte differentiation and a completed epidermal regeneration process. Additionally, the AE + LPPO group showed a significantly reduced number of luminized vessels in the granulation tissue, suggesting a more advanced stage of scar formation.

## 4. Discussion

In the current investigation, we successfully evaluated our idea of combining traditional phytomedicine (based on wound-healing-promoting AE extract) with modern organic chemistry (based on an efficient infection-eradicating molecule LPPO) to create a solid base for a potentially innovative wound-healing-promoting product. Before integrating AE/LPPO into an active dressing, it is essential to comprehend the specific impacts of this combination on wound repair. Our investigation bridges this gap by delving into the mechanisms underlying AE and LPPO’s actions on the in vitro and in vivo levels. Further research revealed that topical application of AE extract, both ethanolic and water-based, significantly reduced wound healing time in rats [30]. Likewise, the topical application of a water-based extract of *Agrimonia pilosa* Ledeb. enhanced epidermal permeability and preserved skin barrier function in mice. Additionally, this extract demonstrated agonistic activity on the transient receptor potential vanilloid 3 (TRPV3) cation channel in keratinocytes [31]. TRPV3, despite being primarily thermosensitive, plays essential roles in keratinocyte proliferation, differentiation, stratum corneum formation [32], hair follicle growth [33], and maintenance of skin barrier function [34]. This knowledge underscores the potential therapeutic significance of *Agrimonia* extracts in wound-healing processes.

The utilization of AE suspension with silver nanoparticles proved successful in the formulation of in situ gels. Interestingly, the antibacterial efficacy against *Staphylococcus aureus* and *Escherichia coli* of the in situ gels surpassed that of silver nanoparticles alone, which exhibited no discernible activity [35]. These findings underscore the promising potential of the in situ gel formulations for application in wound healing therapies. Concurrent antibacterial assays demonstrated that AE, when integrated into a two-layer cotton material coated with poly(vinyl alcohol)-chitosan nanofibers, effectively inhibited the growth of both *S. aureus* and *Pseudomonas aeruginosa* [36]. Additionally, a powdered blend of four herbs, including AE, *Nelumbo nucifera* Gaertn, *Boswellia carteri* Flueck, and *Typha angustifolia*e L., notably enhanced the expression of TGF-β1 and Smad2/3 mRNA during the initial stages of wound healing, while reducing their expression after two weeks [37]. Given our previous findings of AE’s direct influence on the transition from fibroblasts to myofibroblasts in vivo [13], we further investigated the potential underlying mechanisms of action, focusing on its interaction with TGF-β1 signaling [38] through Western blot analysis.

Our previous in vitro study was performed to assess whether LPPO released from PCL-based nanofiber dressing during its degradation impairs selected biological processes associated with wound healing [18]. We demonstrated that LPPO had no negative effect on the proliferation of human dermal fibroblasts, nor did it interfere with the TGF-β1-induced transition from fibroblasts to myofibroblasts or the formation of a dense fibronectin ECM. Specifically, TGF-β1 is transiently upregulated in normal skin wounds, where its role in inducing myofibroblasts is crucial for wound closure [39]. In our experiments, we did not observe any interference by LPPO with TGF-β1 signaling. We also investigated whether LPPO affects the proliferation and differentiation of keratinocytes, the main cells of the epidermis. The assessment of various keratins, along with the significant predominance of E-cadherin over N-cadherin, indicated that keratinocytes maintained a normal state regardless of the presence of LPPO. Furthermore, the capacity of TGF-β1 to either trigger epithelial-to-mesenchymal transition [40] or to inhibit proliferation [41] was also unaffected by LPPO.

Delving into the specific interactions of AE/LPPO co-treatment with signaling pathways reveals intricate modulation across studied cell types. In keratinocytes, while AE/LPPO slightly stimulated the pAKT/pERK signaling pathways, it led to a decrease in the total expression of AKT and ERK without significantly altering the phenotypic changes induced by TGF-*β*1. This suggests that while AE/LPPO can modulate specific signaling pathways, its impact on the overall cellular phenotype, at least in keratinocytes, may be limited. In fibroblasts, we observed an interesting dichotomy where AE/LPPO, in conjunction with TGF-*β*1, increased pAKT expression but reduced total AKT levels. Notably, AE/LPPO did not activate non-canonical pERK signaling regardless of TGF-*β*1 presence, with total ERK being upregulated only in the absence of TGF-*β*1. In endothelial cells, AE/LPPO predominantly stimulated pERK rather than pAKT expression, reinforcing the idea of cell-type-specific responses to AE/LPPO treatment. Despite these cellular effects, co-treatment with AE/LPPO and VEGF did not significantly interfere with VEGF-induced phenotypic changes in endothelial cells, indicating that AE/LPPO’s modulatory effects may not overtly impact VEGF-driven endothelial cell behavior. These findings contribute to our understanding of how AE/LPPO influences signaling pathways in different cell types and underscore the complexity of cellular responses to therapeutic interventions in wound-healing contexts [42].

Another important consideration is the potential antagonism between AE and LPPO in their antibacterial properties. Bioactive compounds can exhibit synergistic or antagonistic effects, with multi-target effects often prevailing. Plant-derived compounds may modify the properties of bioactive constituents, potentially causing overlooked antagonistic interactions resulting in reduced bioavailability of compounds, impacting the extract’s efficacy compared to individual compounds [43,44]. To evaluate possible interaction between the AE extract and LPPO, we performed HPLC analysis, which revealed no or only a very weak interaction (with molecules being released during the HPLC run) between studied compounds. This suggests that any combined effect of the AE extract and LPPO on cells or wound healing is likely to occur at a biological level rather than through direct chemical interaction. Further studies are needed to shed more light on their combined biological activity. For instance, a combination of *Amburana cearensis* A. C. Smith extracts and *Anadenanthera macrocarpa* (Benth.) Brenan with ampicillin produced an antagonistic effect, increasing the MIC from 128 to 512 mg/L [45]. Similarly, an antagonistic relationship was observed between epigallocatechin gallate (EGCg) and glycopeptide antibiotics vancomycin and teicoplanin, suggesting direct binding between EGCg and the antibiotics [46]. Furthermore, a combination of ethanolic extract of *Clinacanthus nutans* (Burm.f.) Lindau and cyclophosphamide exhibited antagonism, potentially linked to alterations in CYP450 enzyme activity or expression [47]. In parallel, our previous findings demonstrated that BSA significantly diminishes the antibacterial activity of LPPO [16], suggesting substantial protein binding akin to compounds like daptomycin [48]. While this may not pose a significant obstacle for topical applications, it remains a noteworthy factor. Moreover, our ongoing advancements in developing new LEGO-LPPO molecules have addressed this issue, as they maintain their activity even in the presence of BSA [49]. Moving forward, we intend to investigate the combination of AE extract with LEGO-LPPO, expecting promising outcomes in our continued research efforts.

Our study, while providing valuable insights into wound healing, has several limitations that warrant consideration. First, the relatively small number of rats utilized may limit the generalizability of our findings, necessitating further studies with larger sample sizes to validate our results. Second, the relatively long evaluation period and the use of a small wound size (4 mm) may not fully capture the complete dynamics of the wound healing process. Third, the absence of an animal model for infected wounds means we could not assess the efficacy of our treatment in the context of infection, a crucial aspect of wound repair. Fourth, the application of a water extract with LPPO falls short of simulating an active wound dressing. Additionally, our research did not investigate the potential molecular interactions between AE/LPPO compounds, which could significantly affect their efficacy, release from a carrier, and mechanism of action in wound healing processes. Of note, the potential antagonism between AE and LPPO leading to a remarkable decrease in antibacterial activity presents another limitation that warrants further investigation. Ultimately, it is not feasible to extrapolate findings from this experimental model to human clinical scenarios because of interspecies variability; however, the overall molecular mechanisms governing wound healing are expected to be comparable. Consequently, any assertions regarding the release and efficacy of active substances for wound healing improvement remain speculative, highlighting the need for future research to develop and test formulations that more closely mimic clinical applications.

## 5. Conclusions

Our study offers compelling evidence of the effects of combining AE extract with the organic compound LPPO in wound healing applications. In vitro analyses demonstrate that AE and LPPO when used together at clinically relevant concentrations, support cell proliferation and ECM production. These findings are strengthened by our in vivo study in the rodent model, which exhibited accelerated wound closure characterized by more mature granulation tissue and a complete process of re-epithelization. However, it is important to note that no positive effect of the AE/LPPO combination was observed in cell migration assays, highlighting that the beneficial outcomes may not extend uniformly across all aspects of wound healing processes. These results emphasize the potential of integrating traditional phytomedicine-based approaches with modern organic chemistry and/or pharmaceutical sciences to innovate wound healing strategies. The study also paves the way for further research aimed at optimizing the delivery and efficacy of AE/LPPO through suitable carrier systems, with the ultimate goal of enhancing wound care in patients. Consequently, our findings provide a solid base for future investigations into the formulation and development of novel wound healing products, promising significant advancements in the treatment and management of wounds.

## 6. Patents

An aqueous solution of *Agrimonia eupatoria* L. and LPPO DR-6180 intended for wound flush has been protected by submitting a utility model application in the Czech Republic under the reg. No. PUV 2024-42308.

## Figures and Tables

**Table 1:** Reagents used for Western blot.

**Primary Antibody**	**Abbreviation**	**Host**	**Isotype**	**Clonality**	**Produced by**
α-smooth muscle actin	SMA	rabbit	IgG	monoclonal	CST, Danvers, MA, USA
Fibronectin	Fibronectin	rabbit	IgG	monoclonal	Abcam, Cambridge, UK
Phospho-ERK1/2	pERK	rabbit		polyclonal	CST, USA
Phospho-AKT	pAKT	rabbit	IgG	monoclonal	CST, USA
Phospho-Smad3	pSmad3	rabbit	IgG	monoclonal	Abcam, UK
ERK1/2	ERK	rabbit	IgG	monoclonal	CST, USA
Smad3	Smad3	rabbit	IgG	monoclonal	CST, USA
AKT	AKT	rabbit		polyclonal	CST, USA
β-actin	β-actin	rabbit	IgG	monoclonal	CST, USA
N-cadherin	N-cadherin	rabbit	IgG	polyclonal	ThermoFisher Scientific, Walthame, MA, USA
CD324(E-cadherin)	E-cadherin	rabbit	IgG1	monoclonal	ThermoFisher Scientific, USA
Cytokeratin 14	Keratin 14	rabbit	IgG	polyclonal	ThermoFisher Scientific, USA
Cytokeratin 19	Keratin 19	mouse	IgG2a	monoclonal	ThermoFisher Scientific, USA
Cytokeratin 8	Keratin 18	mouse	IgG1	monoclonal	ThermoFisher Scientific, USA
VEGF Receptor 2	VEGFR2	rabbit	IgG	monoclonal	CST, USA
Phospho-ERK1/2	pERK	rabbit	IgG	monoclonal	R&D Systems, Minneapolis, MN, USA
Phospho-AKT	pAKT	rabbit	IgG	polyclonal	R&D Systems, USA
ERK1/2	ERK	rabbit	IgG	polyclonal	R&D Systems, USA
AKT	AKT	mouse	IgG2B	monoclonal	R&D Systems, USA
VE-Cadherin	VEC	mouse	IgG2B	monoclonal	R&D Systems, USA
**Secondary Antibody**		**Host**	**Isotype**		**Produced by**
Anti-rabbit, HRP-linked		goat	IgG		CST, USA
Anti-mouse, HRP-linked		horse	IgG		CST, USA
Anti-mouse, HRP-linked		goat	IgG		Invitrogen, Waltham, MN, USA
Anti-rabbit, HRP-linked		goat	IgG		Invitrogen, USA

**Table 2:** Antibodies used for immunofluorescence.

Antibody	Abbreviation	Host	Produced by	Secondary Antibody	Produced by	Channel
α-smooth muscle actin	SMA	Mouse monoclonal	DakoCytomation, Glostrup, Denmark	Goat anti-mouse	Sigma-Aldrich, St. Louis, MO, USA	TRITC-red
Fibronectin	Fibronectin	rabbit polyclonal	DakoCytomation, Glostrup, Denmark	Goat anti-rabbit	Sigma-Aldrich, St. Louis, MO, USA	FITC-green
Cytokeratin 14	Keratin 14	Rabbit polyclonal	ThermoFisher Scientific, USA	Goat anti-rabbit	Sigma-Aldrich, St. Louis, MO, USA	FITC-green
Cytokeratin 19	Keratin 19	Mouse monoclonal	ThermoFisher Scientific, USA	Goat anti-mouse	Sigma-Aldrich, St. Louis, MO, USA	TRITC-red
Cytokeratin 19	Keratin 19	Mouse monoclonal	ThermoFisher Scientific, USA	Goat anti-mouse	ThermoFisher Scientific, USA	TRITC-red
Cytokeratin 8	Keratin 8	Mouse monoclonal	ThermoFisher Scientific, USA	Goat anti-mouse	Sigma-Aldrich, St. Louis, MO, USA	TRITC-red

**Table 3:** Explanation of used scale in the semi-quantitative evaluation of histological sections (ST—surrounding tissue, i.e., tissue out of GT; DL—demarcation line; SCT—subcutaneous tissue; GT—granulation tissue).

Scale	Epithelization	PMNL	Fibroblasts	New Vessels	Collagen
0	thickness of cut edges	absent	absent	absent	absent
1	migration of cells (<50%)	mild ST	mild ST	mild SCT	minimal GT
2	migration of cells (≥50%)	mild DL/GT	mild GT	mild GT	mild GT
3	bridging the excision	moderate DL/GT	moderate GT	moderate GT	moderate GT
4	keratinization	marked DL/GT	marked GT	marked GT	marked GT

**Table 4:** IC_50_ values (µg/mL) of tested *Agrimonia eupatoria* L. extract and lipophosphonoxin (LPPO) DR-6180.

IC_50_	HaCat		HDF		HMVEC	
	without TGF	with TGF	without TGF	with TGF	without VEGF	with VEGF
AE	336,082	286,576	103,269	112,368	289,528	372,921
LPPO	135,360	38,276	38,081	29,120	27,948	29,178

**Table 5:** Results from the semi-quantitative assessment of histological samples.

Parameter: Day/Group:	Epithelization C/LS/AE	PMNL C/LS/AE	Fibroblasts C/LS/AE	Luminized Vessels C/LS/AE	New Collagen C/LS/AE
14	3/3/4	1/0/0	1/1/1	2/2/1	2/2/1

C: untreated control; LS: negative control (moist healing); AE: *Agrimonia eupatoria* L. + LPPO (DR-6180).

## Data Availability

The raw data supporting the conclusions of this article will be made available by the authors upon request.

## Supplementary Materials

The following supporting information can be downloaded at: https://www.mdpi.com/article/10.3390/biom14121590/s1, Figure S1: Scheme of full thickness skin excisions (6-mm in diameter) on the back of each rat.; Figure S2: Wound healing (2D migration)-assay of HaCaT keratinocytes in the presence of *Agrimonia eupatria* L. (AE) water extract (A) and lipophosphonoxin (LPPO) DR-6180 (B). TGF-β1 was used as positive control; Figure S3: Wound healing (2D migration)-assay of HMVEC-d cells in the presence of *Agrimonia eupatria* L. (AE) water extract (A) and lipophosphonoxin (LPPO) DR-6180 (B). VEGF-A was used as positive control; Table S1: Statistics of wound healing-assay of HaCaT keratinocytes in the presence of *Agrimonia eupatria* L. (AE) water extract and lipophosphonoxin (LPPO) DR-6180; Table S2: Statistics of wound healing-assay of HaCaT keratinocytes in the presence of *Agrimonia eupatria* L. (AE) water extract, lipophosphonoxin (LPPO) DR-6180, and TGF-β1; Table S3: Statistics of wound healing-assay of HMVEC-d cells in the presence of *Agrimonia eupatria* L. (AE) water extract and lipophosphonoxin (LPPO) DR-6180; Table S4: Statistics of wound healing-assay of HMVEC-d cells in the presence of *Agrimonia eupatria* L. (AE) water extract, lipophosphonoxin (LPPO) DR-6180, and VEGF-A; Figure S4: Western blot analysis of HaCaT keratinocytes in the presence of *Agrimonia eupatoria* L. (AE) extract, lipophosphonoxin (LPPO) DR-6180 and combination of AE and LPPO. TGF-β1 was used as positive control. Figure S5: Western blot analysis of human dermal fibroblasts (HDF) in the presence of *Agrimonia eupatria* L. (AE) extract, lipophosphonoxin (LPPO) DR-6180 and combination of AE and LPPO. TGF-β1 was used as positive control; Figure S6: Western blot analysis of human dermal microvascular vein endothelial cells (HMVEC-d) in the presence of *Agrimonia eupatria* L. (AE) extract, lipophosphonoxin (LPPO) DR-6180 and combination of AE and LPPO. VEGF-A was used as positive control; Figure S7: Immunofluorescence of HaCaT keratinocytes in the presence of *Agrimonia eupatoria* L. (AE) extract (A), lipophosphonoxin (LPPO) DR-6180 (B), and combination of AE and LPPO (C); Figure S8: Immunofluorescence of human dermal fibroblasts (HDF) in the presence of *Agrimonia eupatoria* L. (AE) extract (A), lipophosphonoxin (LPPO) DR-6180 (B), and combination of AE and LPPO (C).
