# Supplementary material for: Assessment of Agrimonia eupatoria L. and Lipophosphonoxin (DR-6180) Combination for Wound Repair: Bridging the Gap Between Phytomedicine and Organic Chemistry
Source: Biomolecules. 2024 Dec 12;14(12):1590. doi: 10.3390/biom14121590 (PMC11674006; doi:10.3390/biom14121590)
Supplement: Supplementary file 1 [file biomolecules-14-01590-s001.zip › biomolecules-3287609-original-images.pdf]

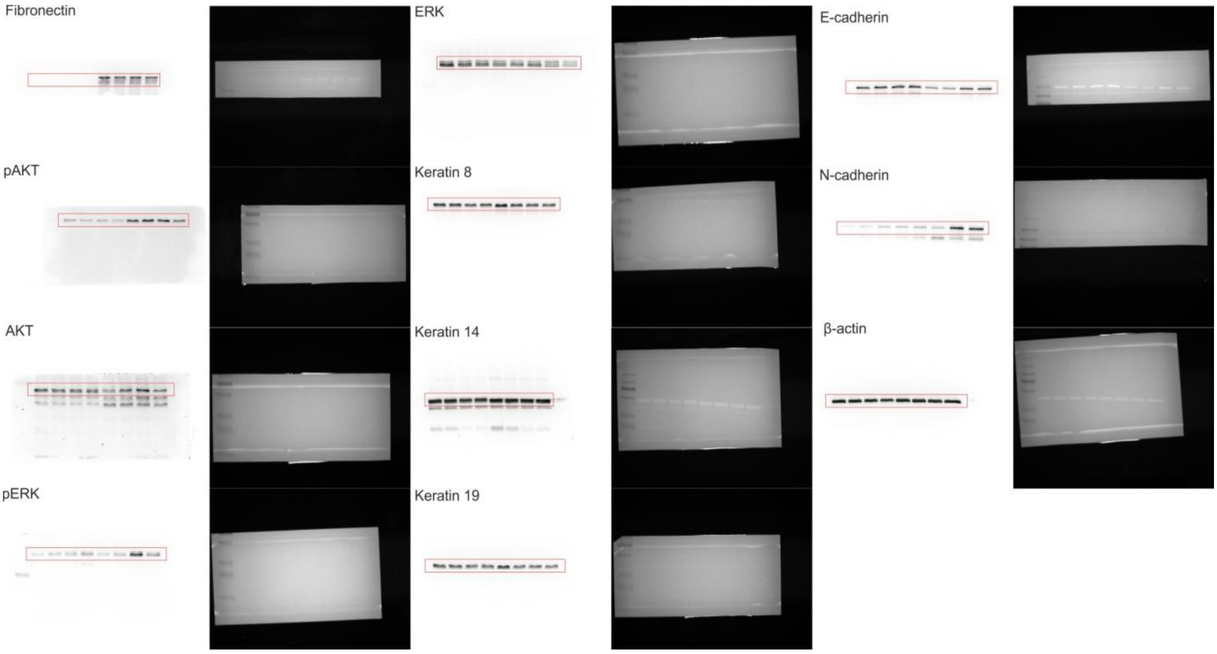

Figure S4. Western blot analysis of HaCaT keratinocytes in the presence of *Agrimonia eupatoria* L. (AE) extract, lipophosphonoxin (LPPO) DR-6180 and combination of AE and LPPO. TGF-β1 was used as positive control.

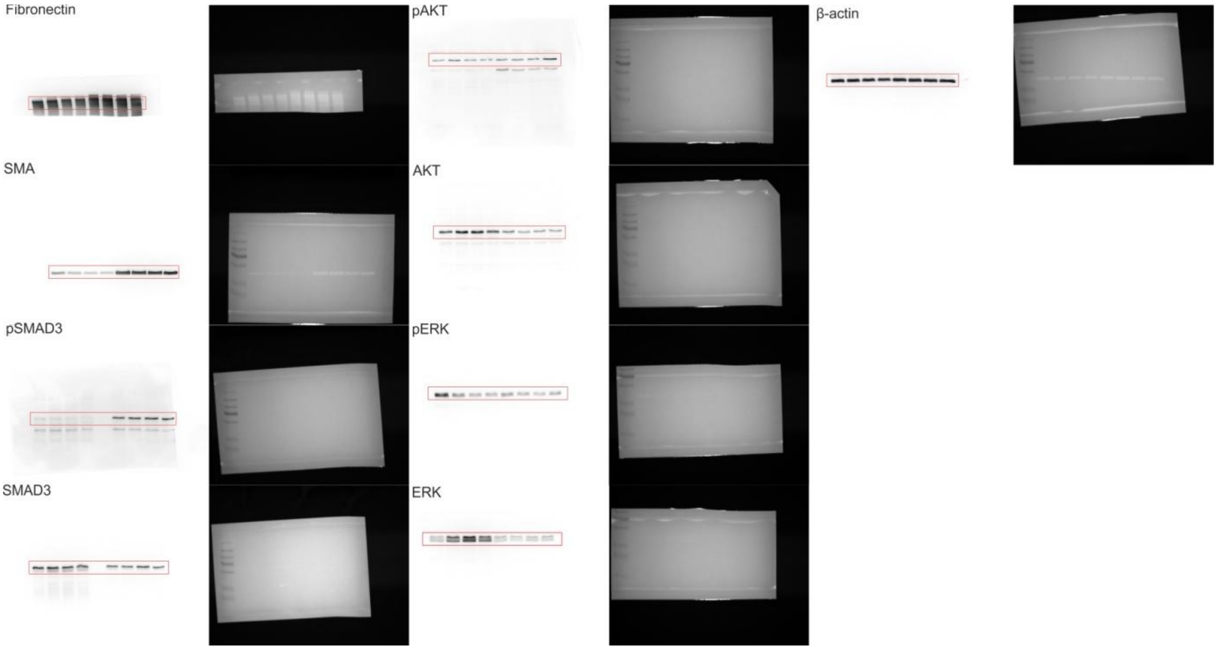

Figure S5. Western blot analysis of human dermal fibroblasts (HDF) in the presence of *Agrimonia eupatria* L. (AE) extract, lipophosphonoxin (LPPO) DR-6180 and combination of AE and LPPO. TGF-β1 was used as positive control.

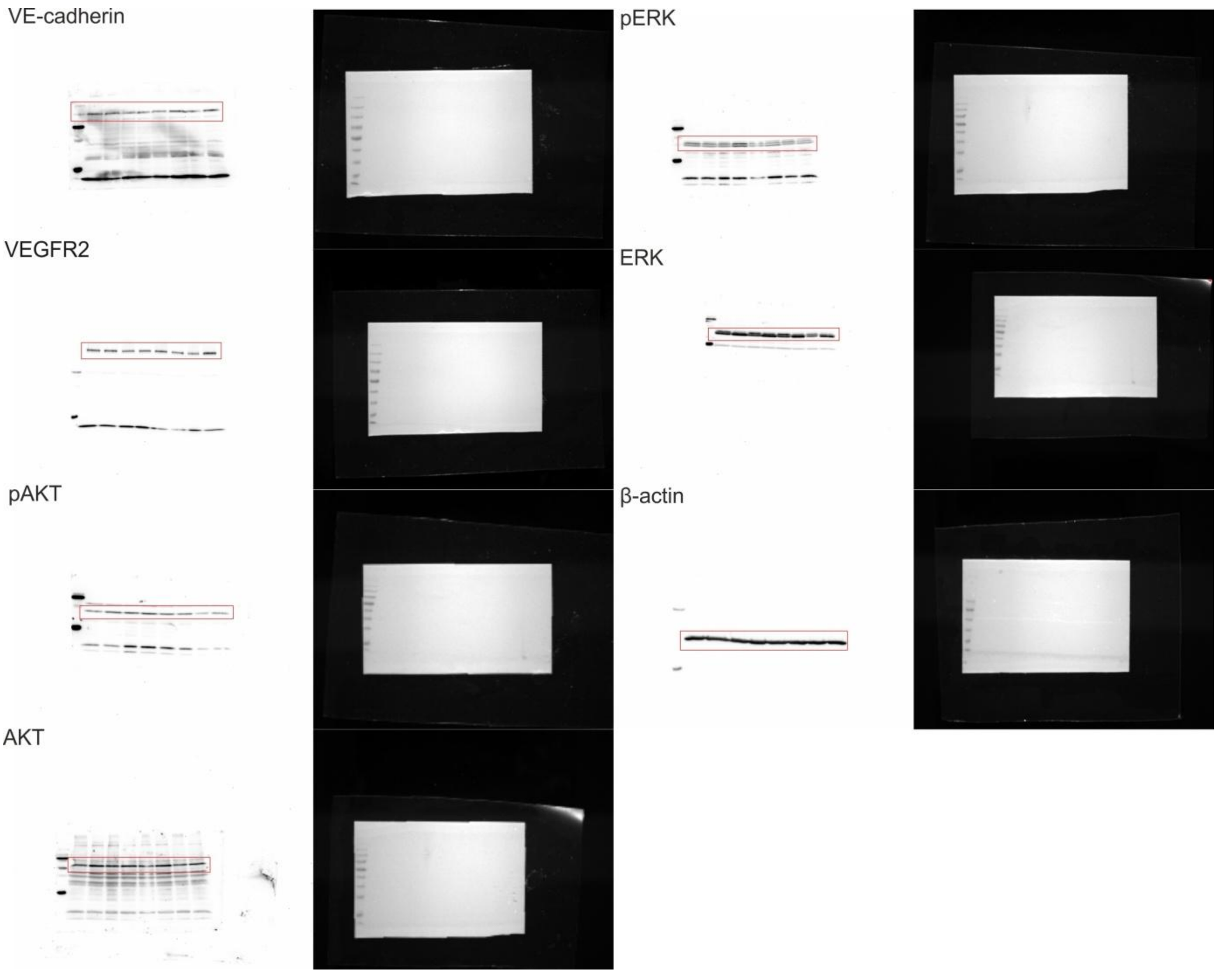

Figure S6. Western blot analysis of human dermal microvascular vein endothelial cells (HMVEC-d) in the presence of *Agrimonia eupatria* L. (AE) extract, lipophosphonoxin (LPPO) DR-6180 and combination of AE and LPPO. VEGF-A was used as positive control.
