# Supplementary material for: Assessment of Agrimonia eupatoria L. and Lipophosphonoxin (DR-6180) Combination for Wound Repair: Bridging the Gap Between Phytomedicine and Organic Chemistry
Source: Biomolecules. 2024 Dec 12;14(12):1590. doi: 10.3390/biom14121590 (PMC11674006; doi:10.3390/biom14121590)
Supplement: Supplementary file 1 [file biomolecules-14-01590-s001.zip › biomolecules-3287609-supplementary.pdf]

<u>Supplementary material:</u> Assessment of *Agrimonia eupatoria* L. and lipophosphonoxin (DR-6180) combination for wound repair: Bridging the gap between phytomedicine and organic chemistry

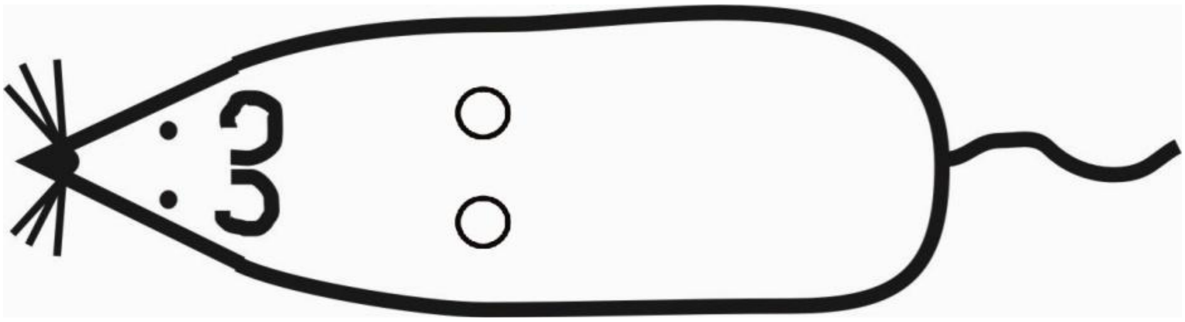

Figure S1. Scheme of full thickness skin excisions (6-mm in diameter) on the back of each rat.

<u>Supplementary material:</u> Assessment of *Agrimonia eupatoria* L. and lipophosphonoxin (DR-6180) combination for wound repair: Bridging the gap between phytomedicine and organic chemistry

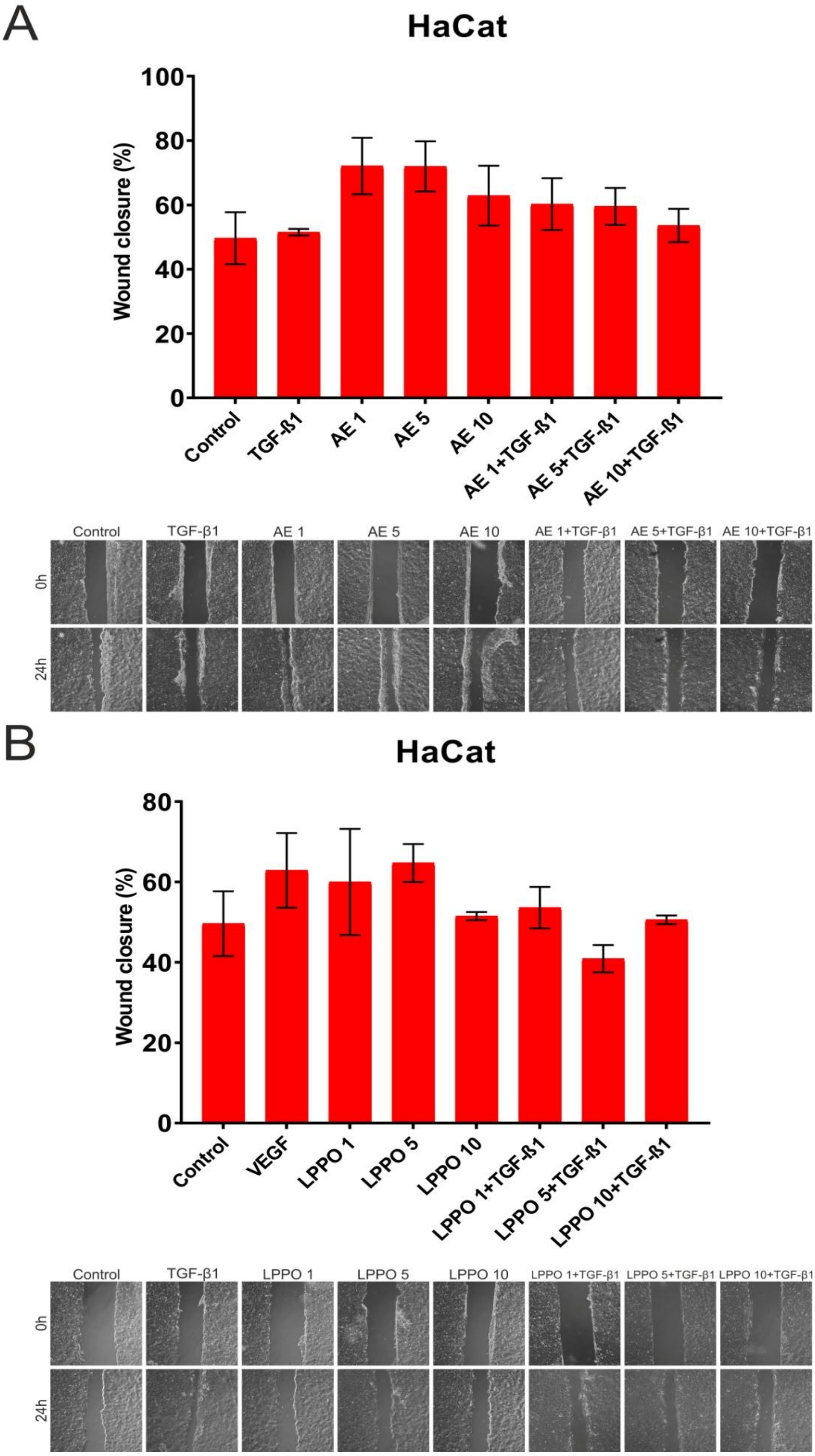

Figure S2. Wound healing (2D migration)-assay of HaCaT keratinocytes in the presence of *Agrimonia eupatria* L. (AE) water extract (A) and lipophosphonoxin (LPPO) DR-6180 (B). TGF-β1 was used as positive control.

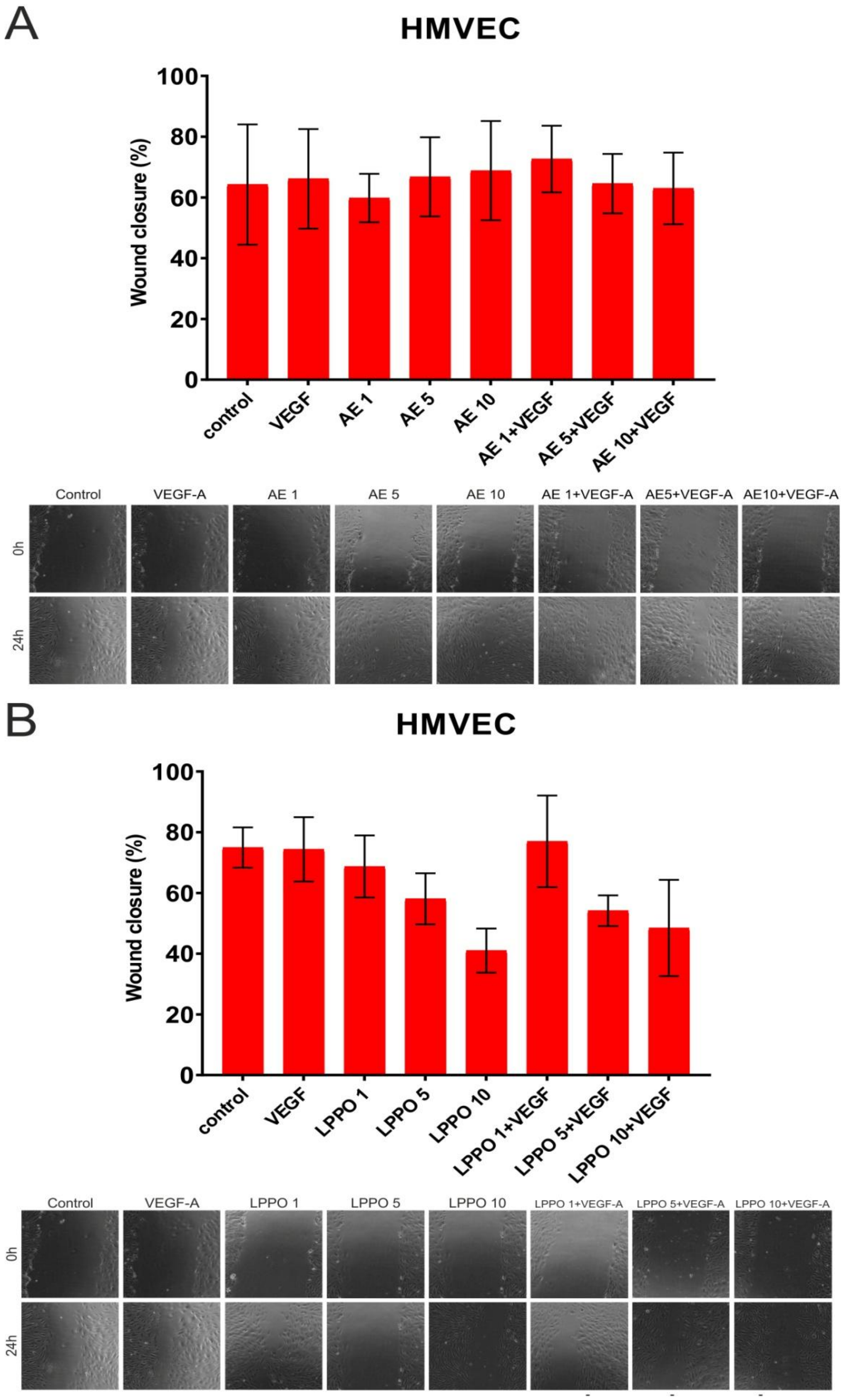

Figure S3. Wound healing (2D migration)-assay of HMVEC-d cells in the presence of *Agrimonia eupatria* L. (AE) water extract (A) and lipophosphonoxin (LPPO) DR-6180 (B). VEGF-A was used as positive control.

<u>Supplementary material:</u> **Assessment of *Agrimonia eupatoria* L. and lipophosphonoxin (DR-6180) combination for wound repair: Bridging the gap between phytomedicine and organic chemistry**

Table S1. Statistics of wound healing-assay of HaCaT keratinocytes in the presence of *Agrimonia eupatria* L. (AE) water extract and lipophosphonoxin (LPPO) DR-6180.

ANOVA

Wound_Closure_Percentage

	Sum of Squares	df	Mean Square	F	Sig.
Between Groups	887.250	9	98.583	1.713	.130
Within Groups	1726.672	30	57.556		
Total	2613.922	39			

Multiple Comparisons (Tukey HSD)

Dependent Variable: Wound_Closure_Percentage

					95% Confidence Interval	
(I) Group	(J) Group	Mean Difference (I-J)	Std. Error	Sig.	Lower Bound	Upper Bound
Control	AE 1+LPPO 1	-13.0832801325	5.3645001450	.340	-31.382571925	5.216011660
	AE 1+LPPO 5	-11.9606330500	5.3645001450	.460	-30.259924842	6.338658742
	AE 1+LPPO 10	-9.2202327900	5.3645001450	.777	-27.519524582	9.079059002
	AE 5+LPPO 1	-11.0711892250	5.3645001450	.565	-29.370481017	7.228102567
	AE 5+LPPO 5	-13.5163893450	5.3645001450	.299	-31.815681137	4.782902447
	AE 5+LPPO 10	-6.8331408900	5.3645001450	.952	-25.132432682	11.466150902
	AE 10+LPPO 1	-17.9136757950	5.3645001450	.059	-36.212967587	.385615997
	AE 10+LPPO 5	-15.0939955850	5.3645001450	.177	-33.393287377	3.205296207
	AE 10+LPPO 10	-8.8229242875	5.3645001450	.816	-27.122216080	9.476367505
AE 1+LPPO 1	Control	13.0832801325	5.3645001450	.340	-5.216011660	31.382571925
	AE 1+LPPO 5	1.1226470825	5.3645001450	1.000	-17.176644710	19.421938875
	AE 1+LPPO 10	3.8630473425	5.3645001450	.999	-14.436244450	22.162339135
	AE 5+LPPO 1	2.0120909075	5.3645001450	1.000	-16.287200885	20.311382700
	AE 5+LPPO 5	-.4331092125	5.3645001450	1.000	-18.732401005	17.866182580
	AE 5+LPPO 10	6.2501392425	5.3645001450	.972	-12.049152550	24.549431035
	AE 10+LPPO 1	-4.8303956625	5.3645001450	.995	-23.129687455	13.468896130
	AE 10+LPPO 5	-2.0107154525	5.3645001450	1.000	-20.310007245	16.288576340
	AE 10+LPPO 10	4.2603558450	5.3645001450	.998	-14.038935947	22.559647637
AE 1+LPPO 5	Control	11.9606330500	5.3645001450	.460	-6.338658742	30.259924842
	AE 1+LPPO 1	-1.1226470825	5.3645001450	1.000	-19.421938875	17.176644710
	AE 1+LPPO 10	2.7404002600	5.3645001450	1.000	-15.558891532	21.039692052
	AE 5+LPPO 1	.8894438250	5.3645001450	1.000	-17.409847967	19.188735617
	AE 5+LPPO 5	-1.5557562950	5.3645001450	1.000	-19.855048087	16.743535497
	AE 5+LPPO 10	5.1274921600	5.3645001450	.993	-13.171799632	23.426783952
	AE 10+LPPO 1	-5.9530427450	5.3645001450	.980	-24.252334537	12.346249047
	AE 10+LPPO 5	-3.1333625350	5.3645001450	1.000	-21.432654327	15.165929257
	AE 10+LPPO 10	3.1377087625	5.3645001450	1.000	-15.161583030	21.437000555
AE 1+LPPO 10	Control	9.2202327900	5.3645001450	.777	-9.079059002	27.519524582

	AE 1+LPPO 1	-3.8630473425	5.3645001450	.999	-22.162339135	14.436244450
	AE 1+LPPO 5	-2.7404002600	5.3645001450	1.000	-21.039692052	15.558891532
	AE 5+LPPO 1	-1.8509564350	5.3645001450	1.000	-20.150248227	16.448335357
	AE 5+LPPO 5	-4.2961565550	5.3645001450	.998	-22.595448347	14.003135237
	AE 5+LPPO 10	2.3870919000	5.3645001450	1.000	-15.912199892	20.686383692
	AE 10+LPPO 1	-8.6934430050	5.3645001450	.828	-26.992734797	9.605848787
	AE 10+LPPO 5	-5.8737627950	5.3645001450	.982	-24.173054587	12.425528997
	AE 10+LPPO 10	.3973085025	5.3645001450	1.000	-17.901983290	18.696600295
AE 5+LPPO 1	Control	11.0711892250	5.3645001450	.565	-7.228102567	29.370481017
	AE 1+LPPO 1	-2.0120909075	5.3645001450	1.000	-20.311382700	16.287200885
	AE 1+LPPO 5	-.8894438250	5.3645001450	1.000	-19.188735617	17.409847967
	AE 1+LPPO 10	1.8509564350	5.3645001450	1.000	-16.448335357	20.150248227
	AE 5+LPPO 5	-2.4452001200	5.3645001450	1.000	-20.744491912	15.854091672
	AE 5+LPPO 10	4.2380483350	5.3645001450	.998	-14.061243457	22.537340127
	AE 10+LPPO 1	-6.8424865700	5.3645001450	.952	-25.141778362	11.456805222
	AE 10+LPPO 5	-4.0228063600	5.3645001450	.999	-22.322098152	14.276485432
	AE 10+LPPO 10	2.2482649375	5.3645001450	1.000	-16.051026855	20.547556730
AE 5+LPPO 5	Control	13.5163893450	5.3645001450	.299	-4.782902447	31.815681137
	AE 1+LPPO 1	.4331092125	5.3645001450	1.000	-17.866182580	18.732401005
	AE 1+LPPO 5	1.5557562950	5.3645001450	1.000	-16.743535497	19.855048087
	AE 1+LPPO 10	4.2961565550	5.3645001450	.998	-14.003135237	22.595448347
	AE 5+LPPO 1	2.4452001200	5.3645001450	1.000	-15.854091672	20.744491912
	AE 5+LPPO 10	6.6832484550	5.3645001450	.958	-11.616043337	24.982540247
	AE 10+LPPO 1	-4.3972864500	5.3645001450	.998	-22.696578242	13.902005342
	AE 10+LPPO 5	-1.5776062400	5.3645001450	1.000	-19.876898032	16.721685552
	AE 10+LPPO 10	4.6934650575	5.3645001450	.996	-13.605826735	22.992756850
AE 5+LPPO 10	Control	6.8331408900	5.3645001450	.952	-11.466150902	25.132432682
	AE 1+LPPO 1	-6.2501392425	5.3645001450	.972	-24.549431035	12.049152550
	AE 1+LPPO 5	-5.1274921600	5.3645001450	.993	-23.426783952	13.171799632
	AE 1+LPPO 10	-2.3870919000	5.3645001450	1.000	-20.686383692	15.912199892
	AE 5+LPPO 1	-4.2380483350	5.3645001450	.998	-22.537340127	14.061243457
	AE 5+LPPO 5	-6.6832484550	5.3645001450	.958	-24.982540247	11.616043337
	AE 10+LPPO 1	-11.0805349050	5.3645001450	.563	-29.379826697	7.218756887
	AE 10+LPPO 5	-8.2608546950	5.3645001450	.865	-26.560146487	10.038437097
	AE 10+LPPO 10	-1.9897833975	5.3645001450	1.000	-20.289075190	16.309508395
AE 10+LPPO 1	Control	17.9136757950	5.3645001450	.059	-.385615997	36.212967587
	AE 1+LPPO 1	4.8303956625	5.3645001450	.995	-13.468896130	23.129687455
	AE 1+LPPO 5	5.9530427450	5.3645001450	.980	-12.346249047	24.252334537
	AE 1+LPPO 10	8.6934430050	5.3645001450	.828	-9.605848787	26.992734797
	AE 5+LPPO 1	6.8424865700	5.3645001450	.952	-11.456805222	25.141778362
	AE 5+LPPO 5	4.3972864500	5.3645001450	.998	-13.902005342	22.696578242

	AE 5+LPPO 10	11.0805349050	5.3645001450	.563	-7.218756887	29.379826697
	AE 10+LPPO 5	2.8196802100	5.3645001450	1.000	-15.479611582	21.118972002
	AE 10+LPPO 10	9.0907515075	5.3645001450	.790	-9.208540285	27.390043300
AE 10+LPPO 5	Control	15.0939955850	5.3645001450	.177	-3.205296207	33.393287377
	AE 1+LPPO 1	2.0107154525	5.3645001450	1.000	-16.288576340	20.310007245
	AE 1+LPPO 5	3.1333625350	5.3645001450	1.000	-15.165929257	21.432654327
	AE 1+LPPO 10	5.8737627950	5.3645001450	.982	-12.425528997	24.173054587
	AE 5+LPPO 1	4.0228063600	5.3645001450	.999	-14.276485432	22.322098152
	AE 5+LPPO 5	1.5776062400	5.3645001450	1.000	-16.721685552	19.876898032
	AE 5+LPPO 10	8.2608546950	5.3645001450	.865	-10.038437097	26.560146487
	AE 10+LPPO 1	-2.8196802100	5.3645001450	1.000	-21.118972002	15.479611582
	AE 10+LPPO 10	6.2710712975	5.3645001450	.972	-12.028220495	24.570363090
AE 10+LPPO 10	Control	8.8229242875	5.3645001450	.816	-9.476367505	27.122216080
	AE 1+LPPO 1	-4.2603558450	5.3645001450	.998	-22.559647637	14.038935947
	AE 1+LPPO 5	-3.1377087625	5.3645001450	1.000	-21.437000555	15.161583030
	AE 1+LPPO 10	-.3973085025	5.3645001450	1.000	-18.696600295	17.901983290
	AE 5+LPPO 1	-2.2482649375	5.3645001450	1.000	-20.547556730	16.051026855
	AE 5+LPPO 5	-4.6934650575	5.3645001450	.996	-22.992756850	13.605826735
	AE 5+LPPO 10	1.9897833975	5.3645001450	1.000	-16.309508395	20.289075190
	AE 10+LPPO 1	-9.0907515075	5.3645001450	.790	-27.390043300	9.208540285
	AE 10+LPPO 5	-6.2710712975	5.3645001450	.972	-24.570363090	12.028220495

Table S2. Statistics of wound healing-assay of HaCaT keratinocytes in the presence of *Agrimonia eupatria* L. (AE) water extract, lipophosphonoxin (LPPO) DR-6180, and TGF-β1.

ANOVA

Wound_Closure_Percentage

	Sum of Squares	df	Mean Square	F	Sig.
Between Groups	393.599	9	43.733	1.989	.077
Within Groups	659.744	30	21.991		
Total	1053.343	39			

Multiple Comparisons (Tukey HSD)

Dependent Variable: Wound_Closure_Percentage

(I) Group	(J) Group	Mean Difference (I-J)	Std. Error	Sig.	95% Confidence Interval	
					Lower Bound	Upper Bound
Control	AE 1+LPPO 1	-2.0407607975	3.3159811063	1.000	-13.352179234	9.270657639
	AE 1+LPPO 5	2.2585111900	3.3159811063	.999	-9.052907246	13.569929626
	AE 1+LPPO 10	-1.0614488025	3.3159811063	1.000	-12.372867239	10.249969634
	AE 5+LPPO 1	-.9053810025	3.3159811063	1.000	-12.216799439	10.406037434
	AE 5+LPPO 5	5.1368505850	3.3159811063	.861	-6.174567851	16.448269021
	AE 5+LPPO 10	8.4277998250	3.3159811063	.288	-2.883618611	19.739218261
	AE 10+LPPO 1	1.6092618925	3.3159811063	1.000	-9.702156544	12.920680329
	AE 10+LPPO 5	.9032236575	3.3159811063	1.000	-10.408194779	12.214642094
	AE 10+LPPO 10	4.7853312300	3.3159811063	.903	-6.526087206	16.096749666
AE 1+LPPO 1	Control	2.0407607975	3.3159811063	1.000	-9.270657639	13.352179234
	AE 1+LPPO 5	4.2992719875	3.3159811063	.947	-7.012146449	15.610690424
	AE 1+LPPO 10	.9793119950	3.3159811063	1.000	-10.332106441	12.290730431
	AE 5+LPPO 1	1.1353797950	3.3159811063	1.000	-10.176038641	12.446798231
	AE 5+LPPO 5	7.1776113825	3.3159811063	.500	-4.133807054	18.489029819
	AE 5+LPPO 10	10.4685606225	3.3159811063	.088	-.842857814	21.779979059
	AE 10+LPPO 1	3.6500226900	3.3159811063	.981	-7.661395746	14.961441126
	AE 10+LPPO 5	2.9439844550	3.3159811063	.996	-8.367433981	14.255402891
	AE 10+LPPO 10	6.8260920275	3.3159811063	.568	-4.485326409	18.137510464
AE 1+LPPO 5	Control	-2.2585111900	3.3159811063	.999	-13.569929626	9.052907246
	AE 1+LPPO 1	-4.2992719875	3.3159811063	.947	-15.610690424	7.012146449
	AE 1+LPPO 10	-3.3199599925	3.3159811063	.990	-14.631378429	7.991458444
	AE 5+LPPO 1	-3.1638921925	3.3159811063	.993	-14.475310629	8.147526244
	AE 5+LPPO 5	2.8783393950	3.3159811063	.996	-8.433079041	14.189757831
	AE 5+LPPO 10	6.1692886350	3.3159811063	.694	-5.142129801	17.480707071
	AE 10+LPPO 1	-.6492492975	3.3159811063	1.000	-11.960667734	10.662169139
	AE 10+LPPO 5	-1.3552875325	3.3159811063	1.000	-12.666705969	9.956130904
	AE 10+LPPO 10	2.5268200400	3.3159811063	.999	-8.784598396	13.838238476
AE 1+LPPO 10	Control	1.0614488025	3.3159811063	1.000	-10.249969634	12.372867239

	AE 1+LPPO 1	-.9793119950	3.3159811063	1.000	-12.290730431	10.332106441
	AE 1+LPPO 5	3.3199599925	3.3159811063	.990	-7.991458444	14.631378429
	AE 5+LPPO 1	.1560678000	3.3159811063	1.000	-11.155350636	11.467486236
	AE 5+LPPO 5	6.1982993875	3.3159811063	.688	-5.113119049	17.509717824
	AE 5+LPPO 10	9.4892486275	3.3159811063	.161	-1.822169809	20.800667064
	AE 10+LPPO 1	2.6707106950	3.3159811063	.998	-8.640707741	13.982129131
	AE 10+LPPO 5	1.9646724600	3.3159811063	1.000	-9.346745976	13.276090896
	AE 10+LPPO 10	5.8467800325	3.3159811063	.752	-5.464638404	17.158198469
AE 5+LPPO 1	Control	.9053810025	3.3159811063	1.000	-10.406037434	12.216799439
	AE 1+LPPO 1	-1.1353797950	3.3159811063	1.000	-12.446798231	10.176038641
	AE 1+LPPO 5	3.1638921925	3.3159811063	.993	-8.147526244	14.475310629
	AE 1+LPPO 10	-.1560678000	3.3159811063	1.000	-11.467486236	11.155350636
	AE 5+LPPO 5	6.0422315875	3.3159811063	.717	-5.269186849	17.353650024
	AE 5+LPPO 10	9.3331808275	3.3159811063	.177	-1.978237609	20.644599264
	AE 10+LPPO 1	2.5146428950	3.3159811063	.999	-8.796775541	13.826061331
	AE 10+LPPO 5	1.8086046600	3.3159811063	1.000	-9.502813776	13.120023096
	AE 10+LPPO 10	5.6907122325	3.3159811063	.778	-5.620706204	17.002130669
AE 5+LPPO 5	Control	-5.1368505850	3.3159811063	.861	-16.448269021	6.174567851
	AE 1+LPPO 1	-7.1776113825	3.3159811063	.500	-18.489029819	4.133807054
	AE 1+LPPO 5	-2.8783393950	3.3159811063	.996	-14.189757831	8.433079041
	AE 1+LPPO 10	-6.1982993875	3.3159811063	.688	-17.509717824	5.113119049
	AE 5+LPPO 1	-6.0422315875	3.3159811063	.717	-17.353650024	5.269186849
	AE 5+LPPO 10	3.2909492400	3.3159811063	.991	-8.020469196	14.602367676
	AE 10+LPPO 1	-3.5275886925	3.3159811063	.985	-14.839007129	7.783829744
	AE 10+LPPO 5	-4.2336269275	3.3159811063	.951	-15.545045364	7.077791509
	AE 10+LPPO 10	-.3515193550	3.3159811063	1.000	-11.662937791	10.959899081
AE 5+LPPO 10	Control	-8.4277998250	3.3159811063	.288	-19.739218261	2.883618611
	AE 1+LPPO 1	-10.4685606225	3.3159811063	.088	-21.779979059	.842857814
	AE 1+LPPO 5	-6.1692886350	3.3159811063	.694	-17.480707071	5.142129801
	AE 1+LPPO 10	-9.4892486275	3.3159811063	.161	-20.800667064	1.822169809
	AE 5+LPPO 1	-9.3331808275	3.3159811063	.177	-20.644599264	1.978237609
	AE 5+LPPO 5	-3.2909492400	3.3159811063	.991	-14.602367676	8.020469196
	AE 10+LPPO 1	-6.8185379325	3.3159811063	.569	-18.129956369	4.492880504
	AE 10+LPPO 5	-7.5245761675	3.3159811063	.436	-18.835994604	3.786842269
	AE 10+LPPO 10	-3.6424685950	3.3159811063	.981	-14.953887031	7.668949841
AE 10+LPPO 1	Control	-1.6092618925	3.3159811063	1.000	-12.920680329	9.702156544
	AE 1+LPPO 1	-3.6500226900	3.3159811063	.981	-14.961441126	7.661395746
	AE 1+LPPO 5	.6492492975	3.3159811063	1.000	-10.662169139	11.960667734
	AE 1+LPPO 10	-2.6707106950	3.3159811063	.998	-13.982129131	8.640707741
	AE 5+LPPO 1	-2.5146428950	3.3159811063	.999	-13.826061331	8.796775541
	AE 5+LPPO 5	3.5275886925	3.3159811063	.985	-7.783829744	14.839007129

	AE 5+LPPO 10	6.8185379325	3.3159811063	.569	-4.492880504	18.129956369
	AE 10+LPPO 5	-.7060382350	3.3159811063	1.000	-12.017456671	10.605380201
	AE 10+LPPO 10	3.1760693375	3.3159811063	.993	-8.135349099	14.487487774
AE 10+LPPO 5	Control	-.9032236575	3.3159811063	1.000	-12.214642094	10.408194779
	AE 1+LPPO 1	-2.9439844550	3.3159811063	.996	-14.255402891	8.367433981
	AE 1+LPPO 5	1.3552875325	3.3159811063	1.000	-9.956130904	12.666705969
	AE 1+LPPO 10	-1.9646724600	3.3159811063	1.000	-13.276090896	9.346745976
	AE 5+LPPO 1	-1.8086046600	3.3159811063	1.000	-13.120023096	9.502813776
	AE 5+LPPO 5	4.2336269275	3.3159811063	.951	-7.077791509	15.545045364
	AE 5+LPPO 10	7.5245761675	3.3159811063	.436	-3.786842269	18.835994604
	AE 10+LPPO 1	.7060382350	3.3159811063	1.000	-10.605380201	12.017456671
	AE 10+LPPO 10	3.8821075725	3.3159811063	.971	-7.429310864	15.193526009
AE 10+LPPO 10	Control	-4.7853312300	3.3159811063	.903	-16.096749666	6.526087206
	AE 1+LPPO 1	-6.8260920275	3.3159811063	.568	-18.137510464	4.485326409
	AE 1+LPPO 5	-2.5268200400	3.3159811063	.999	-13.838238476	8.784598396
	AE 1+LPPO 10	-5.8467800325	3.3159811063	.752	-17.158198469	5.464638404
	AE 5+LPPO 1	-5.6907122325	3.3159811063	.778	-17.002130669	5.620706204
	AE 5+LPPO 5	.3515193550	3.3159811063	1.000	-10.959899081	11.662937791
	AE 5+LPPO 10	3.6424685950	3.3159811063	.981	-7.668949841	14.953887031
	AE 10+LPPO 1	-3.1760693375	3.3159811063	.993	-14.487487774	8.135349099
	AE 10+LPPO 5	-3.8821075725	3.3159811063	.971	-15.193526009	7.429310864

Supplementary material: Assessment of *Agrimonia eupatoria* L. and lipophosphonoxin (DR-6180) combination for wound repair: Bridging the gap between phytomedicine and organic chemistry

Table S3. Statistics of wound healing-assay of HMVEC-d cells in the presence of *Agrimonia eupatria* L. (AE) water extract and lipophosphonoxin (LPPO) DR-6180.

ANOVA

Wound_Closure_Percentage

	Sum of Squares	df	Mean Square	F	Sig.
Between Groups	1043.460	9	115.940	1.490	.178
Within Groups	3890.018	50	77.800		
Total	4933.479	59			

Multiple Comparisons (Tukey HSD)

Dependent Variable: Wound_Closure_Percentage

Tukey HSD

(I) Group	(J) Group	Mean Difference (I-J)	Std. Error	Sig.	95% Confidence Interval	
					Lower Bound	Upper Bound
Control	AE 1+LPPO 1	-8.6000261933	5.0924900721	.796	-25.457545151	8.257492764
	AE 1+LPPO 5	-3.1148192000	5.0924900721	1.000	-19.972338157	13.742699757
	AE 1+LPPO 10	3.2691666550	5.0924900721	1.000	-13.588352302	20.126685612
	AE 5+LPPO 1	-4.5106367733	5.0924900721	.996	-21.368155731	12.346882184
	AE 5+LPPO 5	-3.7856307100	5.0924900721	.999	-20.643149667	13.071888247
	AE 5+LPPO 10	-5.8792322550	5.0924900721	.976	-22.736751212	10.978286702
	AE 10+LPPO 1	-8.3322213467	5.0924900721	.824	-25.189740304	8.525297611
	AE 10+LPPO 5	-4.9939153900	5.0924900721	.992	-21.851434347	11.863603567
	AE 10+LPPO 10	4.1388834267	5.0924900721	.998	-12.718635531	20.996402384
AE 1+LPPO 1	Control	8.6000261933	5.0924900721	.796	-8.257492764	25.457545151
	AE 1+LPPO 5	5.4852069933	5.0924900721	.985	-11.372311964	22.342725951
	AE 1+LPPO 10	11.8691928483	5.0924900721	.388	-4.988326109	28.726711806
	AE 5+LPPO 1	4.0893894200	5.0924900721	.998	-12.768129537	20.946908377
	AE 5+LPPO 5	4.8143954833	5.0924900721	.994	-12.043123474	21.671914441
	AE 5+LPPO 10	2.7207939383	5.0924900721	1.000	-14.136725019	19.578312896
	AE 10+LPPO 1	.2678048467	5.0924900721	1.000	-16.589714111	17.125323804
	AE 10+LPPO 5	3.6061108033	5.0924900721	.999	-13.251408154	20.463629761
	AE 10+LPPO 10	12.7389096200	5.0924900721	.293	-4.118609337	29.596428577
AE 1+LPPO 5	Control	3.1148192000	5.0924900721	1.000	-13.742699757	19.972338157
	AE 1+LPPO 1	-5.4852069933	5.0924900721	.985	-22.342725951	11.372311964
	AE 1+LPPO 10	6.3839858550	5.0924900721	.959	-10.473533102	23.241504812
	AE 5+LPPO 1	-1.3958175733	5.0924900721	1.000	-18.253336531	15.461701384
	AE 5+LPPO 5	-.6708115100	5.0924900721	1.000	-17.528330467	16.186707447
	AE 5+LPPO 10	-2.7644130550	5.0924900721	1.000	-19.621932012	14.093105902
	AE 10+LPPO 1	-5.2174021467	5.0924900721	.989	-22.074921104	11.640116811
	AE 10+LPPO 5	-1.8790961900	5.0924900721	1.000	-18.736615147	14.978422767
	AE 10+LPPO 10	7.2537026267	5.0924900721	.914	-9.603816331	24.111221584
AE 1+LPPO 10	Control	-3.2691666550	5.0924900721	1.000	-20.126685612	13.588352302

	AE 1+LPPO 1	-11.8691928483	5.0924900721	.388	-28.726711806	4.988326109
	AE 1+LPPO 5	-6.3839858550	5.0924900721	.959	-23.241504812	10.473533102
	AE 5+LPPO 1	-7.7798034283	5.0924900721	.874	-24.637322386	9.077715529
	AE 5+LPPO 5	-7.0547973650	5.0924900721	.926	-23.912316322	9.802721592
	AE 5+LPPO 10	-9.1483989100	5.0924900721	.734	-26.005917867	7.709120047
	AE 10+LPPO 1	-11.6013880017	5.0924900721	.421	-28.458906959	5.256130956
	AE 10+LPPO 5	-8.2630820450	5.0924900721	.831	-25.120601002	8.594436912
	AE 10+LPPO 10	.8697167717	5.0924900721	1.000	-15.987802186	17.727235729
AE 5+LPPO 1	Control	4.5106367733	5.0924900721	.996	-12.346882184	21.368155731
	AE 1+LPPO 1	-4.0893894200	5.0924900721	.998	-20.946908377	12.768129537
	AE 1+LPPO 5	1.3958175733	5.0924900721	1.000	-15.461701384	18.253336531
	AE 1+LPPO 10	7.7798034283	5.0924900721	.874	-9.077715529	24.637322386
	AE 5+LPPO 5	.7250060633	5.0924900721	1.000	-16.132512894	17.582525021
	AE 5+LPPO 10	-1.3685954817	5.0924900721	1.000	-18.226114439	15.488923476
	AE 10+LPPO 1	-3.8215845733	5.0924900721	.999	-20.679103531	13.035934384
	AE 10+LPPO 5	-.4832786167	5.0924900721	1.000	-17.340797574	16.374240341
	AE 10+LPPO 10	8.6495202000	5.0924900721	.791	-8.207998757	25.507039157
AE 5+LPPO 5	Control	3.7856307100	5.0924900721	.999	-13.071888247	20.643149667
	AE 1+LPPO 1	-4.8143954833	5.0924900721	.994	-21.671914441	12.043123474
	AE 1+LPPO 5	.6708115100	5.0924900721	1.000	-16.186707447	17.528330467
	AE 1+LPPO 10	7.0547973650	5.0924900721	.926	-9.802721592	23.912316322
	AE 5+LPPO 1	-.7250060633	5.0924900721	1.000	-17.582525021	16.132512894
	AE 5+LPPO 10	-2.0936015450	5.0924900721	1.000	-18.951120502	14.763917412
	AE 10+LPPO 1	-4.5465906367	5.0924900721	.996	-21.404109594	12.310928321
	AE 10+LPPO 5	-1.2082846800	5.0924900721	1.000	-18.065803637	15.649234277
	AE 10+LPPO 10	7.9245141367	5.0924900721	.862	-8.933004821	24.782033094
AE 5+LPPO 10	Control	5.8792322550	5.0924900721	.976	-10.978286702	22.736751212
	AE 1+LPPO 1	-2.7207939383	5.0924900721	1.000	-19.578312896	14.136725019
	AE 1+LPPO 5	2.7644130550	5.0924900721	1.000	-14.093105902	19.621932012
	AE 1+LPPO 10	9.1483989100	5.0924900721	.734	-7.709120047	26.005917867
	AE 5+LPPO 1	1.3685954817	5.0924900721	1.000	-15.488923476	18.226114439
	AE 5+LPPO 5	2.0936015450	5.0924900721	1.000	-14.763917412	18.951120502
	AE 10+LPPO 1	-2.4529890917	5.0924900721	1.000	-19.310508049	14.404529866
	AE 10+LPPO 5	.8853168650	5.0924900721	1.000	-15.972202092	17.742835822
	AE 10+LPPO 10	10.0181156817	5.0924900721	.625	-6.839403276	26.875634639
AE 10+LPPO 1	Control	8.3322213467	5.0924900721	.824	-8.525297611	25.189740304
	AE 1+LPPO 1	-.2678048467	5.0924900721	1.000	-17.125323804	16.589714111
	AE 1+LPPO 5	5.2174021467	5.0924900721	.989	-11.640116811	22.074921104
	AE 1+LPPO 10	11.6013880017	5.0924900721	.421	-5.256130956	28.458906959
	AE 5+LPPO 1	3.8215845733	5.0924900721	.999	-13.035934384	20.679103531
	AE 5+LPPO 5	4.5465906367	5.0924900721	.996	-12.310928321	21.404109594

	AE 5+LPPO 10	2.4529890917	5.0924900721	1.000	-14.404529866	19.310508049
	AE 10+LPPO 5	3.3383059567	5.0924900721	1.000	-13.519213001	20.195824914
	AE 10+LPPO 10	12.4711047733	5.0924900721	.321	-4.386414184	29.328623731
AE 10+LPPO 5	Control	4.9939153900	5.0924900721	.992	-11.863603567	21.851434347
	AE 1+LPPO 1	-3.6061108033	5.0924900721	.999	-20.463629761	13.251408154
	AE 1+LPPO 5	1.8790961900	5.0924900721	1.000	-14.978422767	18.736615147
	AE 1+LPPO 10	8.2630820450	5.0924900721	.831	-8.594436912	25.120601002
	AE 5+LPPO 1	.4832786167	5.0924900721	1.000	-16.374240341	17.340797574
	AE 5+LPPO 5	1.2082846800	5.0924900721	1.000	-15.649234277	18.065803637
	AE 5+LPPO 10	-.8853168650	5.0924900721	1.000	-17.742835822	15.972202092
	AE 10+LPPO 1	-3.3383059567	5.0924900721	1.000	-20.195824914	13.519213001
	AE 10+LPPO 10	9.1327988167	5.0924900721	.736	-7.724720141	25.990317774
AE 10+LPPO 10	Control	-4.1388834267	5.0924900721	.998	-20.996402384	12.718635531
	AE 1+LPPO 1	-12.7389096200	5.0924900721	.293	-29.596428577	4.118609337
	AE 1+LPPO 5	-7.2537026267	5.0924900721	.914	-24.111221584	9.603816331
	AE 1+LPPO 10	-.8697167717	5.0924900721	1.000	-17.727235729	15.987802186
	AE 5+LPPO 1	-8.6495202000	5.0924900721	.791	-25.507039157	8.207998757
	AE 5+LPPO 5	-7.9245141367	5.0924900721	.862	-24.782033094	8.933004821
	AE 5+LPPO 10	-10.0181156817	5.0924900721	.625	-26.875634639	6.839403276
	AE 10+LPPO 1	-12.4711047733	5.0924900721	.321	-29.328623731	4.386414184
	AE 10+LPPO 5	-9.1327988167	5.0924900721	.736	-25.990317774	7.724720141

Table S4. Statistics of wound healing-assay of HMVEC-d cells in the presence of *Agrimonia eupatria* L. (AE) water extract, lipophosphonoxin (LPPO) DR-6180, and VEGF-A.

ANOVA

Wound_Closure_Percentage

	Sum of Squares	df	Mean Square	F	Sig.
Between Groups	1527.796	9	169.755	1.265	.280
Within Groups	6576.832	49	134.221		
Total	8104.628	58			

Multiple Comparisons (Tukey HSD)

Dependent Variable: Wound_Closure_Percentage

(I) Group	(J) Group	Mean Difference (I-J)	Std. Error	Sig.	95% Confidence Interval	
					Lower Bound	Upper Bound
Control	AE 1+LPPO 1	-7.4697708200	6.6888228942	.981	-29.631987624	14.692445984
	AE 1+LPPO 5	2.3360143983	6.6888228942	1.000	-19.826202406	24.498231203
	AE 1+LPPO 10	13.0393774933	6.6888228942	.637	-9.122839311	35.201594298
	AE 5+LPPO 1	3.6252544217	6.6888228942	1.000	-18.536962383	25.787471226
	AE 5+LPPO 5	5.1189323233	6.6888228942	.999	-17.043284481	27.281149128
	AE 5+LPPO 10	7.7879384400	6.6888228942	.974	-14.374278364	29.950155244
	AE 10+LPPO 1	4.6402958450	6.6888228942	.999	-17.521920959	26.802512649
	AE 10+LPPO 5	5.9168911400	6.6888228942	.996	-16.245325664	28.079107944
	AE 10+LPPO 10	5.8331293717	7.0152966353	.998	-17.410799708	29.077058451
AE 1+LPPO 1	Control	7.4697708200	6.6888228942	.981	-14.692445984	29.631987624
	AE 1+LPPO 5	9.8057852183	6.6888228942	.899	-12.356431586	31.968002023
	AE 1+LPPO 10	20.5091483133	6.6888228942	.092	-1.653068491	42.671365118
	AE 5+LPPO 1	11.0950252417	6.6888228942	.812	-11.067191563	33.257242046
	AE 5+LPPO 5	12.5887031433	6.6888228942	.681	-9.573513661	34.750919948
	AE 5+LPPO 10	15.2577092600	6.6888228942	.419	-6.904507544	37.419926064
	AE 10+LPPO 1	12.1100666650	6.6888228942	.726	-10.052150139	34.272283469
	AE 10+LPPO 5	13.3866619600	6.6888228942	.602	-8.775554844	35.548878764
	AE 10+LPPO 10	13.3029001917	7.0152966353	.671	-9.941028888	36.546829271
AE 1+LPPO 5	Control	-2.3360143983	6.6888228942	1.000	-24.498231203	19.826202406
	AE 1+LPPO 1	-9.8057852183	6.6888228942	.899	-31.968002023	12.356431586
	AE 1+LPPO 10	10.7033630950	6.6888228942	.842	-11.458853709	32.865579899
	AE 5+LPPO 1	1.2892400233	6.6888228942	1.000	-20.872976781	23.451456828
	AE 5+LPPO 5	2.7829179250	6.6888228942	1.000	-19.379298879	24.945134729
	AE 5+LPPO 10	5.4519240417	6.6888228942	.998	-16.710292763	27.614140846
	AE 10+LPPO 1	2.3042814467	6.6888228942	1.000	-19.857935358	24.466498251
	AE 10+LPPO 5	3.5808767417	6.6888228942	1.000	-18.581340063	25.743093546
	AE 10+LPPO 10	3.4971149733	7.0152966353	1.000	-19.746814106	26.741044053
AE 1+LPPO	Control	-13.0393774933	6.6888228942	.637	-35.201594298	9.122839311

10	AE 1+LPPO 1	-20.5091483133	6.6888228942	.092	-42.671365118	1.653068491
	AE 1+LPPO 5	-10.7033630950	6.6888228942	.842	-32.865579899	11.458853709
	AE 5+LPPO 1	-9.4141230717	6.6888228942	.919	-31.576339876	12.748093733
	AE 5+LPPO 5	-7.9204451700	6.6888228942	.971	-30.082661974	14.241771634
	AE 5+LPPO 10	-5.2514390533	6.6888228942	.999	-27.413655858	16.910777751
	AE 10+LPPO 1	-8.3990816483	6.6888228942	.959	-30.561298453	13.763135156
	AE 10+LPPO 5	-7.1224863533	6.6888228942	.986	-29.284703158	15.039730451
	AE 10+LPPO 10	-7.2062481217	7.0152966353	.989	-30.450177201	16.037680958
AE 5+LPPO 1	Control	-3.6252544217	6.6888228942	1.000	-25.787471226	18.536962383
	AE 1+LPPO 1	-11.0950252417	6.6888228942	.812	-33.257242046	11.067191563
	AE 1+LPPO 5	-1.2892400233	6.6888228942	1.000	-23.451456828	20.872976781
	AE 1+LPPO 10	9.4141230717	6.6888228942	.919	-12.748093733	31.576339876
	AE 5+LPPO 5	1.4936779017	6.6888228942	1.000	-20.668538903	23.655894706
	AE 5+LPPO 10	4.1626840183	6.6888228942	1.000	-17.999532786	26.324900823
	AE 10+LPPO 1	1.0150414233	6.6888228942	1.000	-21.147175381	23.177258228
	AE 10+LPPO 5	2.2916367183	6.6888228942	1.000	-19.870580086	24.453853523
	AE 10+LPPO 10	2.2078749500	7.0152966353	1.000	-21.036054129	25.451804029
AE 5+LPPO 5	Control	-5.1189323233	6.6888228942	.999	-27.281149128	17.043284481
	AE 1+LPPO 1	-12.5887031433	6.6888228942	.681	-34.750919948	9.573513661
	AE 1+LPPO 5	-2.7829179250	6.6888228942	1.000	-24.945134729	19.379298879
	AE 1+LPPO 10	7.9204451700	6.6888228942	.971	-14.241771634	30.082661974
	AE 5+LPPO 1	-1.4936779017	6.6888228942	1.000	-23.655894706	20.668538903
	AE 5+LPPO 10	2.6690061167	6.6888228942	1.000	-19.493210688	24.831222921
	AE 10+LPPO 1	-.4786364783	6.6888228942	1.000	-22.640853283	21.683580326
	AE 10+LPPO 5	.7979588167	6.6888228942	1.000	-21.364257988	22.960175621
	AE 10+LPPO 10	.7141970483	7.0152966353	1.000	-22.529732031	23.958126128
AE 5+LPPO 10	Control	-7.7879384400	6.6888228942	.974	-29.950155244	14.374278364
	AE 1+LPPO 1	-15.2577092600	6.6888228942	.419	-37.419926064	6.904507544
	AE 1+LPPO 5	-5.4519240417	6.6888228942	.998	-27.614140846	16.710292763
	AE 1+LPPO 10	5.2514390533	6.6888228942	.999	-16.910777751	27.413655858
	AE 5+LPPO 1	-4.1626840183	6.6888228942	1.000	-26.324900823	17.999532786
	AE 5+LPPO 5	-2.6690061167	6.6888228942	1.000	-24.831222921	19.493210688
	AE 10+LPPO 1	-3.1476425950	6.6888228942	1.000	-25.309859399	19.014574209
	AE 10+LPPO 5	-1.8710473000	6.6888228942	1.000	-24.033264104	20.291169504
	AE 10+LPPO 10	-1.9548090683	7.0152966353	1.000	-25.198738148	21.289120011
AE 10+LPPO 1	Control	-4.6402958450	6.6888228942	.999	-26.802512649	17.521920959
	AE 1+LPPO 1	-12.1100666650	6.6888228942	.726	-34.272283469	10.052150139
	AE 1+LPPO 5	-2.3042814467	6.6888228942	1.000	-24.466498251	19.857935358
	AE 1+LPPO 10	8.3990816483	6.6888228942	.959	-13.763135156	30.561298453
	AE 5+LPPO 1	-1.0150414233	6.6888228942	1.000	-23.177258228	21.147175381
	AE 5+LPPO 5	.4786364783	6.6888228942	1.000	-21.683580326	22.640853283

	AE 5+LPPO 10	3.1476425950	6.6888228942	1.000	-19.014574209	25.309859399
	AE 10+LPPO 5	1.2765952950	6.6888228942	1.000	-20.885621509	23.438812099
	AE 10+LPPO 10	1.1928335267	7.0152966353	1.000	-22.051095553	24.436762606
AE 10+LPPO 5	Control	-5.9168911400	6.6888228942	.996	-28.079107944	16.245325664
	AE 1+LPPO 1	-13.3866619600	6.6888228942	.602	-35.548878764	8.775554844
	AE 1+LPPO 5	-3.5808767417	6.6888228942	1.000	-25.743093546	18.581340063
	AE 1+LPPO 10	7.1224863533	6.6888228942	.986	-15.039730451	29.284703158
	AE 5+LPPO 1	-2.2916367183	6.6888228942	1.000	-24.453853523	19.870580086
	AE 5+LPPO 5	-.7979588167	6.6888228942	1.000	-22.960175621	21.364257988
	AE 5+LPPO 10	1.8710473000	6.6888228942	1.000	-20.291169504	24.033264104
	AE 10+LPPO 1	-1.2765952950	6.6888228942	1.000	-23.438812099	20.885621509
	AE 10+LPPO 10	-.0837617683	7.0152966353	1.000	-23.327690848	23.160167311
AE 10+LPPO 10	Control	-5.8331293717	7.0152966353	.998	-29.077058451	17.410799708
	AE 1+LPPO 1	-13.3029001917	7.0152966353	.671	-36.546829271	9.941028888
	AE 1+LPPO 5	-3.4971149733	7.0152966353	1.000	-26.741044053	19.746814106
	AE 1+LPPO 10	7.2062481217	7.0152966353	.989	-16.037680958	30.450177201
	AE 5+LPPO 1	-2.2078749500	7.0152966353	1.000	-25.451804029	21.036054129
	AE 5+LPPO 5	-.7141970483	7.0152966353	1.000	-23.958126128	22.529732031
	AE 5+LPPO 10	1.9548090683	7.0152966353	1.000	-21.289120011	25.198738148
	AE 10+LPPO 1	-1.1928335267	7.0152966353	1.000	-24.436762606	22.051095553
	AE 10+LPPO 5	.0837617683	7.0152966353	1.000	-23.160167311	23.327690848

Supplementary material: **Assessment of *Agrimonia eupatoria* L. and lipophosphonoxin (DR-6180) combination for wound repair: Bridging the gap between phytomedicine and organic chemistry**

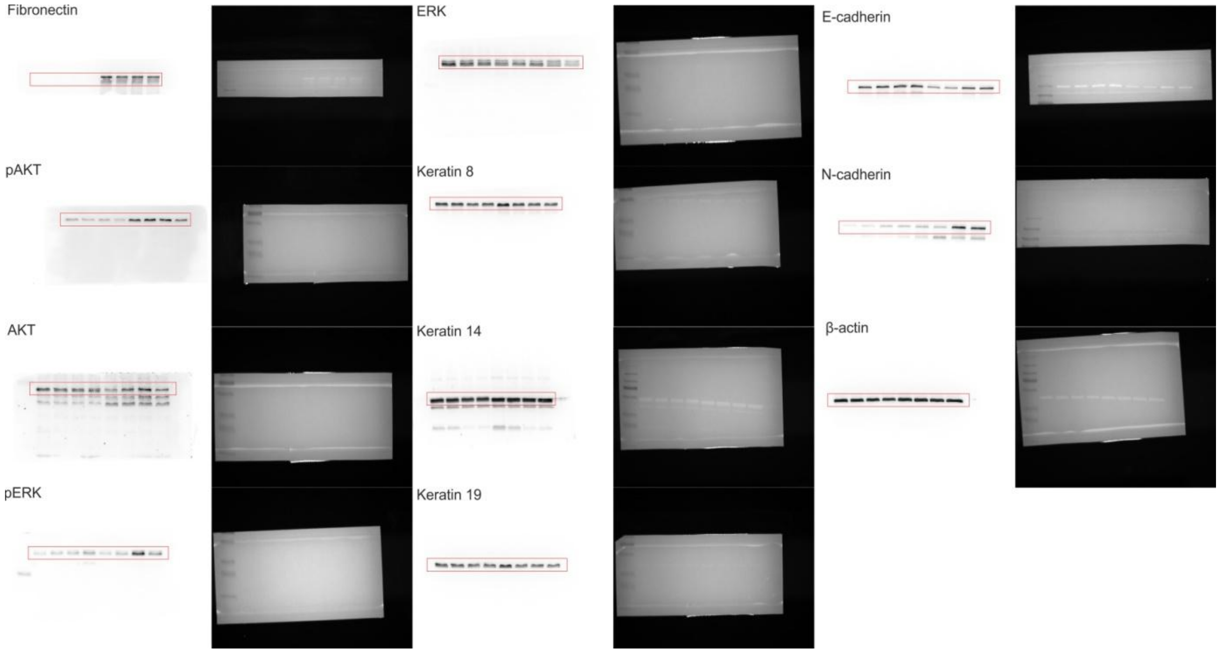

Figure S4. Western blot analysis of HaCaT keratinocytes in the presence of *Agrimonia eupatoria* L. (AE) extract, lipophosphonoxin (LPPO) DR-6180 and combination of AE and LPPO. TGF-β1 was used as positive control.

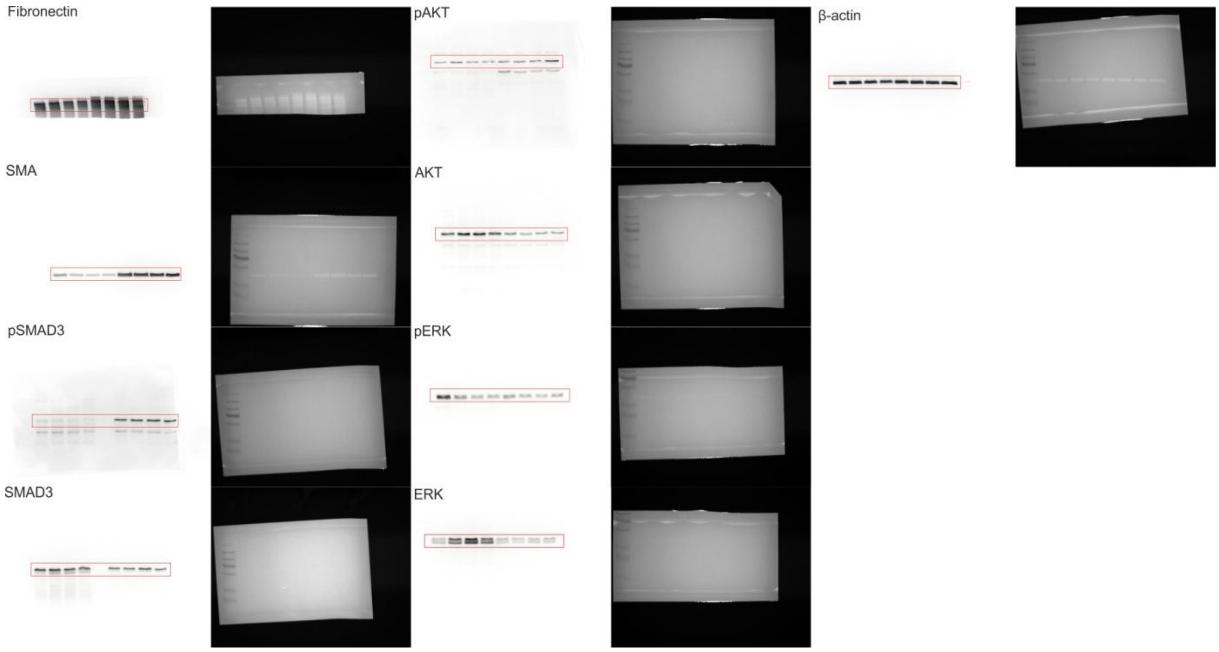

Figure S5. Western blot analysis of human dermal fibroblasts (HDF) in the presence of *Agrimonia eupatria* L. (AE) extract, lipophosphonoxin (LPPO) DR-6180 and combination of AE and LPPO. TGF-β1 was used as positive control.

<u>Supplementary material:</u> Assessment of *Agrimonia eupatoria* L. and lipophosphonoxin (DR-6180) combination for wound repair: Bridging the gap between phytomedicine and organic chemistry

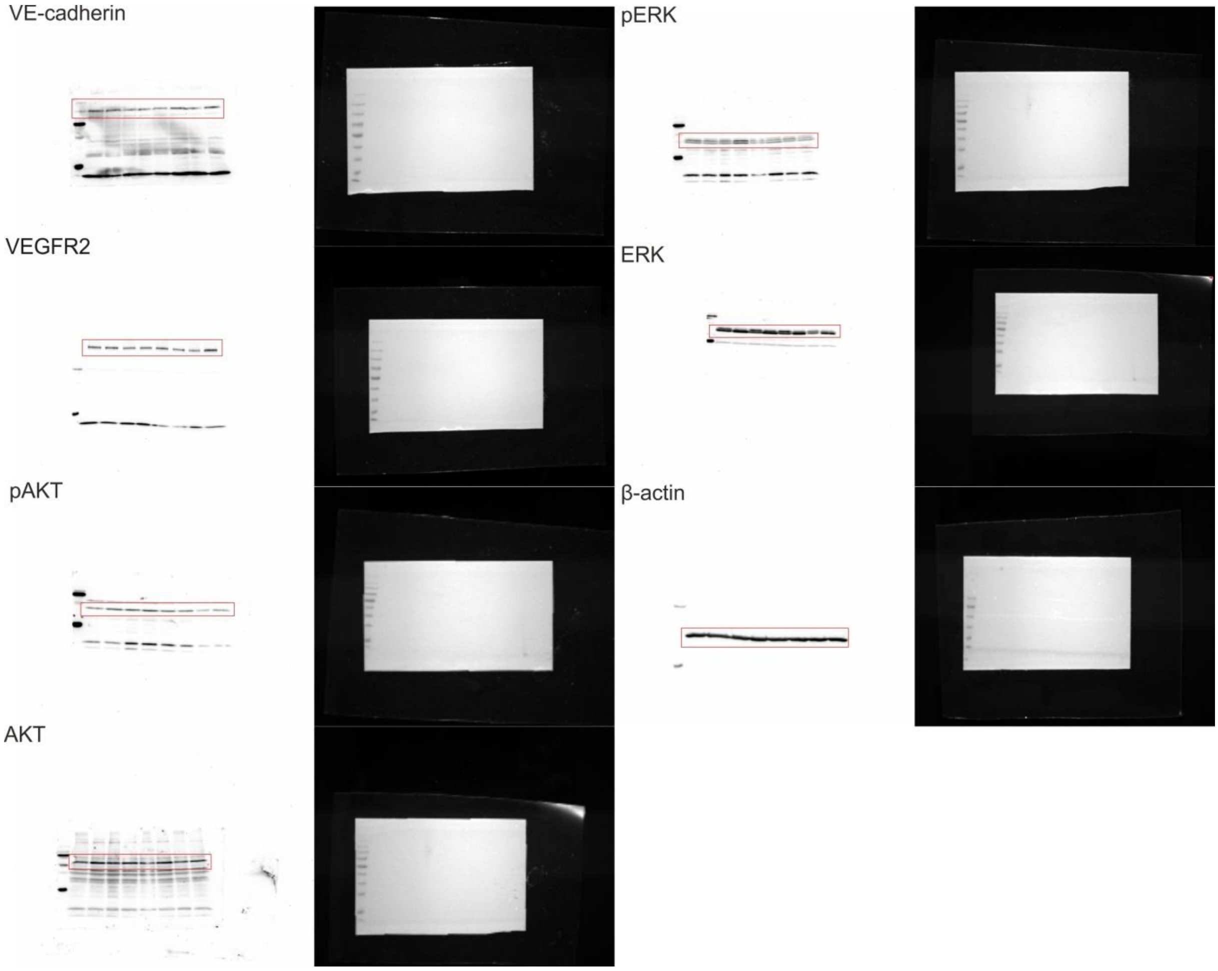

Figure S6. Western blot analysis of human dermal microvascular vein endothelial cells (HMVEC-d) in the presence of *Agrimonia eupatria* L. (AE) extract, lipophosphonoxin (LPPO) DR-6180 and combination of AE and LPPO. VEGF-A was used as positive control.

Supplementary material: **Assessment of *Agrimonia eupatoria* L. and lipophosphonoxin (DR-6180) combination for wound repair: Bridging the gap between phytomedicine and organic chemistry**

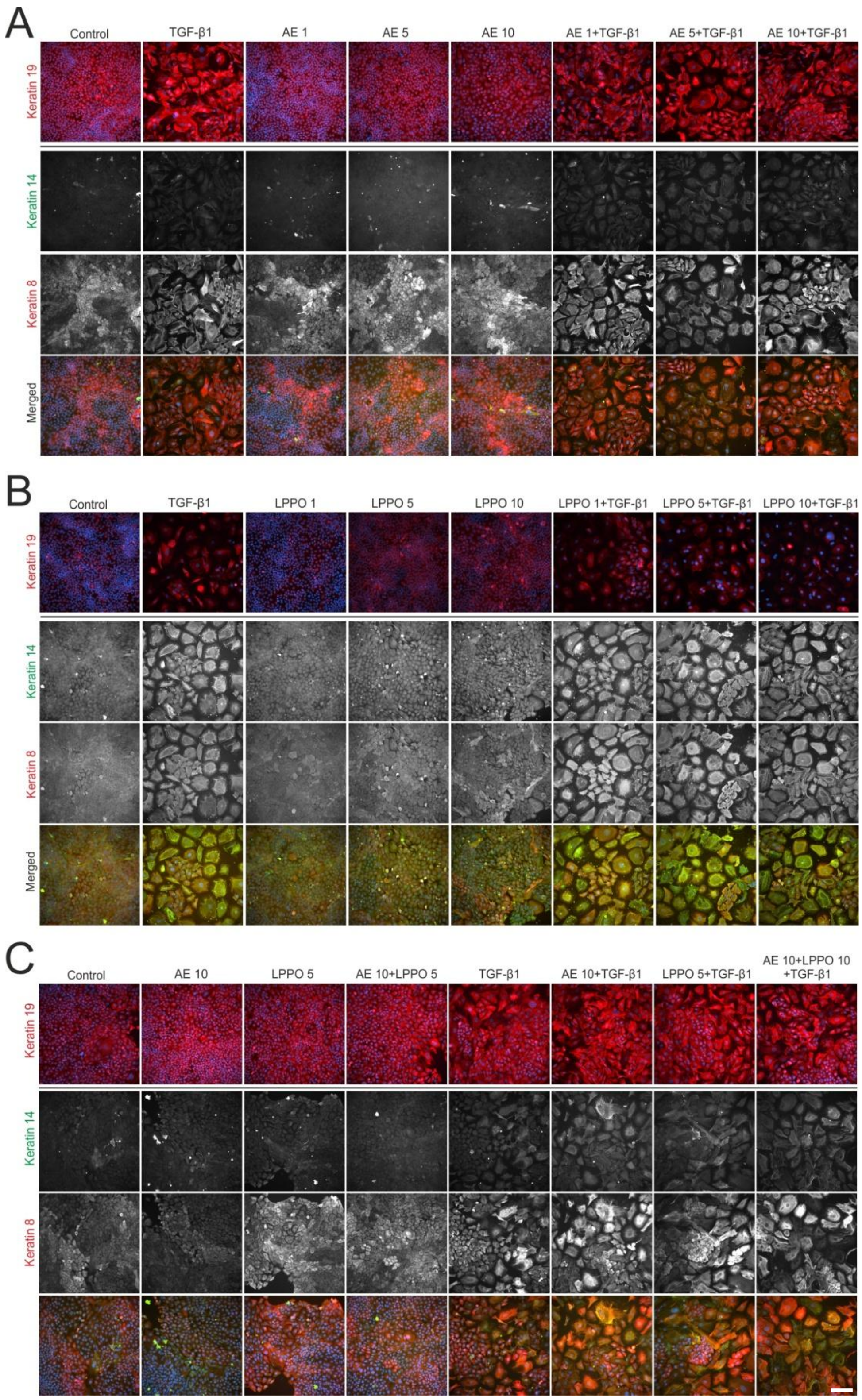

Figure S7. Immunofluorescence of HaCaT keratinocytes in the presence of *Agrimonia eupatoria* L. (AE) extract (A), lipophosphonoxin (LPPO) DR-6180 (B), and combination of AE and LPPO (C). TGF-β1 was used as the positive control. Magnification 200x; scale bar = 100 μm.

Supplementary material: **Assessment of *Agrimonia eupatoria* L. and lipophosphonoxin (DR-6180) combination for wound repair: Bridging the gap between phytomedicine and organic chemistry**

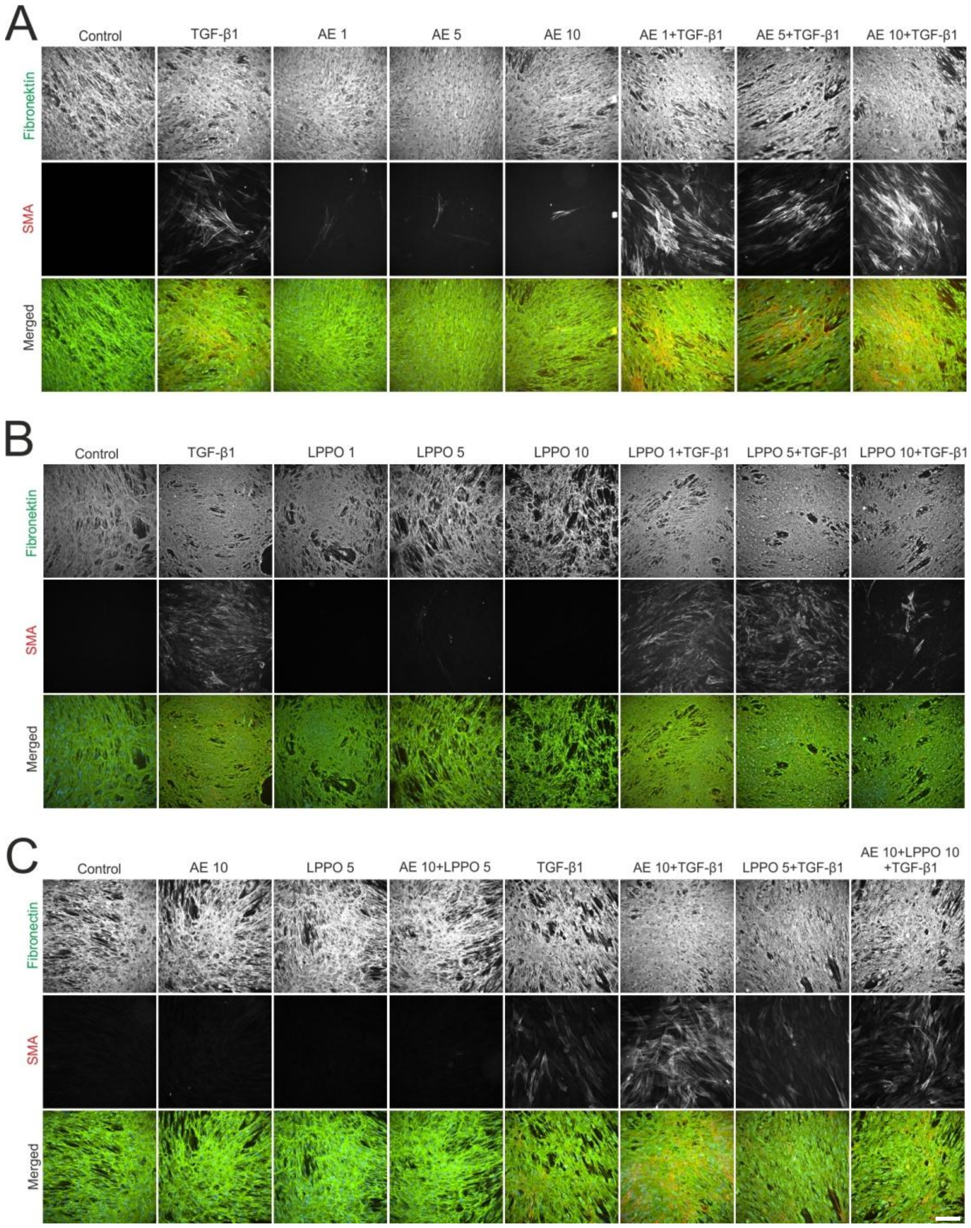

Figure S8. Immunofluorescence of human dermal fibroblasts (HDF) in the presence of *Agrimonia eupatoria* L. (AE) extract (A), lipophosphonoxin (LPPO) DR-6180 (B), and combination of AE and LPPO (C). TGF-β1 was used as the positive control. Magnification 200x; scale bar = 100 μm.
